# Enterohemorrhagic *Escherichia coli* senses low biotin status in the large intestine for colonization and infection

**DOI:** 10.1038/ncomms7592

**Published:** 2015-03-20

**Authors:** Bin Yang, Lu Feng, Fang Wang, Lei Wang

**Affiliations:** 1 TEDA Institute of Biological Sciences and Biotechnology, Nankai University, TEDA, Tianjin 300457, P.R. China; 2 Key Laboratory of Molecular Microbiology and Technology, Ministry of Education, Tianjin 300071, P.R. China; 3 Tianjin Key Laboratory of Microbial Functional Genomics, Tianjin 300457, P.R. China; 4 State Key Laboratory of Medicinal Chemical Biology, Nankai University, Tianjin 300071, P.R. China; 5 SynBio Research Platform, Collaborative Innovation Center of Chemical Science and Engineering (Tianjin), Tianjin 300072, P.R. China

## Abstract

Enterohemorrhagic *Escherichia coli* (EHEC) is an important foodborne pathogen that infects humans by colonizing the large intestine. Here we identify a virulence-regulating pathway in which the biotin protein ligase BirA signals to the global regulator Fur, which in turn activates LEE (locus of enterocyte effacement) genes to promote EHEC adherence in the low-biotin large intestine. LEE genes are repressed in the high-biotin small intestine, thus preventing adherence and ensuring selective colonization of the large intestine. The presence of this pathway in all nine EHEC serotypes tested indicates that it is an important evolutionary strategy for EHEC. The pathway is incomplete in closely related small-intestinal enteropathogenic *E. coli* due to the lack of the Fur response to BirA. Mice fed with a biotin-rich diet show significantly reduced EHEC adherence, indicating that biotin might be useful to prevent EHEC infection in humans.

## Abstract (web summary)

Enterohaemorrhagic *Escherichia coli* (EHEC) is an important foodborne pathogen that colonizes the large intestine. Here, the authors identify a signalling pathway that controls EHEC adherence to host cells in response to variations in biotin levels, ensuring selective colonization of the large intestine.

## Graphical abstract

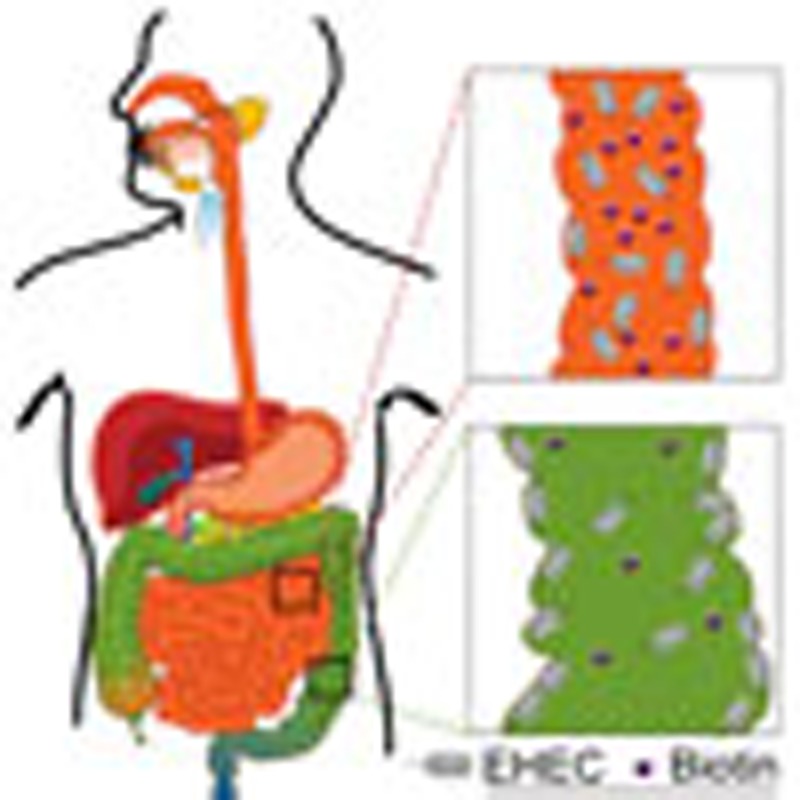

Enteropathogenic *Escherichia coli* (EPEC) and enterohemorrhagic *E. coli* (EHEC) are important human gastrointestinal pathogens. EPEC causes watery diarrhoea, mainly in children in developing countries^1^. EHEC induces much severer symptoms, producing diarrhoea complicated by haemorrhagic colitis and, sometimes, haemolytic uremic syndrome, which is fatal in 3–75% of cases^2^. Among the survivors, 30% show chronic renal failure, hypertension and neurological damage^2^. The two pathotypes differ in two major virulence features: the ability of EHEC and inability of EPEC to produce Shiga toxins; and the colonization of the large intestine by EHEC versus the small intestine by EPEC^3^. Although the molecular basis of Shiga toxins and their implications for pathogenicity have been extensively investigated^4^, the mechanisms behind the site-specific colonization of these two closely related pathotypes are still not well known.

The establishment of successful colonization, characterized by the formation of attaching and effacing (A/E) lesions, is critical for EHEC and EPEC to cause infection because both bacteria are noninvasive pathogens^3^. The ability to form A/E lesions is conferred by the locus of enterocyte effacement (LEE), which consists of five polycistronic operons (LEE1 to LEE5) that are conserved in all A/E-forming pathogens^3^,^5^. LEE1 to LEE3 encode a type III secretion system that exports effector molecules; LEE5 encodes the outer membrane adhesin intimin and the translocated intimin receptor (Tir), which are necessary for intimate attachment to the host epithelium; and LEE4 encodes additional type III secretion system structural components, translocator and effector proteins^3^,^5^. Distinct intimin types have been identified^6^. Intimin γ-expressing EHEC adhered to distal ileal Peyer’s patches, whereas intimin α-expressing EPEC adhered to both Peyer’s patches and other small intestinal explants based on *in vitro* human intestinal organ culture adhesion assays^7^,^8^,^9^,^10^. Replacement of intimin γ with intimin α in EHEC O157:H7 produces an intimin α-like tropism^11^. Site-directed mutagenesis of the receptor-binding site of intimin can also influence bacterial tissue tropism^12^. However, these results cannot fully explain the pathotype-specific tissue tropism between EHEC and EPEC. The initial adherence to host cells may also be promoted by other adhesins, but none of these adhesins were found to be related to the site-specific colonization of EHEC and EPEC^13^.

The expression of LEE genes is regulated by a range of global and specific regulators and is influenced by different environmental stimuli^14^. The human small and large intestines represent two different environments. Several environmental factors present in the human gastrointestinal tract have been found to affect the expression of LEE genes or adherence of EHEC and EPEC by *in vitro* assays^14^,^15^,^16^,^17^,^18^. For example, low-pH stress enhanced the adhesion of EHEC to epithelial cells^16^. Adherence to host cells and/or secretion of LEE-encoded virulence factors by EPEC is enhanced by low-pH stress and bile-salt stress^17^ and repressed by ammonium^18^. However, bile-salt treatment had no effect on LEE gene expression in EHEC^19^. Thus, whether these factors are linked to the site-specific colonization of EHEC and EPEC needs to be examined further.

Biotin functions as an essential cofactor for carboxylases and decarboxylases in all organisms. This cofactor can either be synthesized *de novo* or taken up by microorganisms^20^. In contrast, humans can only obtain biotin from external sources mainly through small intestinal absorption; therefore, higher biotin levels are expected in the small intestine compared with the large intestine^20^,^21^. In *E. coli*, biotin synthesis is tightly controlled in response to biotin supply/demand via the biotin protein ligase BirA, which acts as both the enzyme that funnels biotin into the metabolism and a negative transcriptional regulator of the biotin synthetic operon (*bio* operon), containing *bioA* and *bioBFCD*^20^,^22^. When biotin levels exceed bacterial physiological needs, BirA in complex with biotin binds directly to the 40-bp biotin operator (*bioO*) to repress the transcription of the *bio* operon^23^. BirA homologues (known as holocarboxylase synthetase) are also present in humans and play regulatory roles in biotin metabolism and transport^20^,^22^. The N-terminal domain of BirA forms a winged helix–turn–helix motif that is common to many global regulatory proteins^24^. However, no involvement of this biotin-dependent regulator in non-biotin-related processes has been reported.

EHEC O157:H7 (O157), the most notorious EHEC strain due to its high mortality rate, is thought to have evolved from the less virulent EPEC O55:H7 (O55)^25^. The close relationship between this pair of bacteria makes them suitable for use in comparative studies of the differences in EHEC and EPEC virulence. Genomic comparisons have revealed that genetic determinants acquired during the evolutionary process contribute to the higher virulence of O157:H7, especially Shiga toxin-converting phages and a large virulence plasmid (pO157)^26^. However, these analyses provided no hints concerning the molecular basis of the different colonization sites of the bacteria. The expression of LEE genes, which are present in identical copies in the two strains, in response to different conditions in the small and large intestines have not been compared.

In this study, we investigate possible mechanisms underlying the different adherence abilities of EHEC and EPEC. We compare the gene expression profiles of EHEC O157 and EPEC O55 after 3 h of incubation with HeLa cells using the RNA-Seq technique. *In silico* analysis through comparative bioinformatics, and *in situ* functional characterization through quantitative reverse transcription–PCR (qRT–PCR) and mutation assays, reveal the presence of a BirA-mediated biotin signalling regulatory pathway that acts via Fur to control LEE gene expression and bacterial adherence in O157 but not O55. LEE gene expression is positively regulated by Fur in both O157 and O55. Chromatin immunoprecipitation (ChIP) sequencing (ChIP-Seq) analysis is used to screen possible BirA-binding sites, and ChIP-quantitative PCR (qPCR) and electrophoretic mobility shift (EMSA) assays confirm the binding of BirA to the *fur* promoter. There is a negative correlation between O157 adherence and biotin concentrations in the small and large intestines of mice, and reduced O157 adherence is observed in mice fed a high-biotin diet. This regulatory pathway is present in an additional 19 EHEC strains but absent in 11 EPEC strains. We conclude that this regulatory pathway is used for the site-specific colonization of the large intestine by EHEC, and propose that biotin is potentially useful for the prevention of EHEC infection.

## Results

### O157 adheres to HeLa epithelial cells better than O55

**Figure 1:**
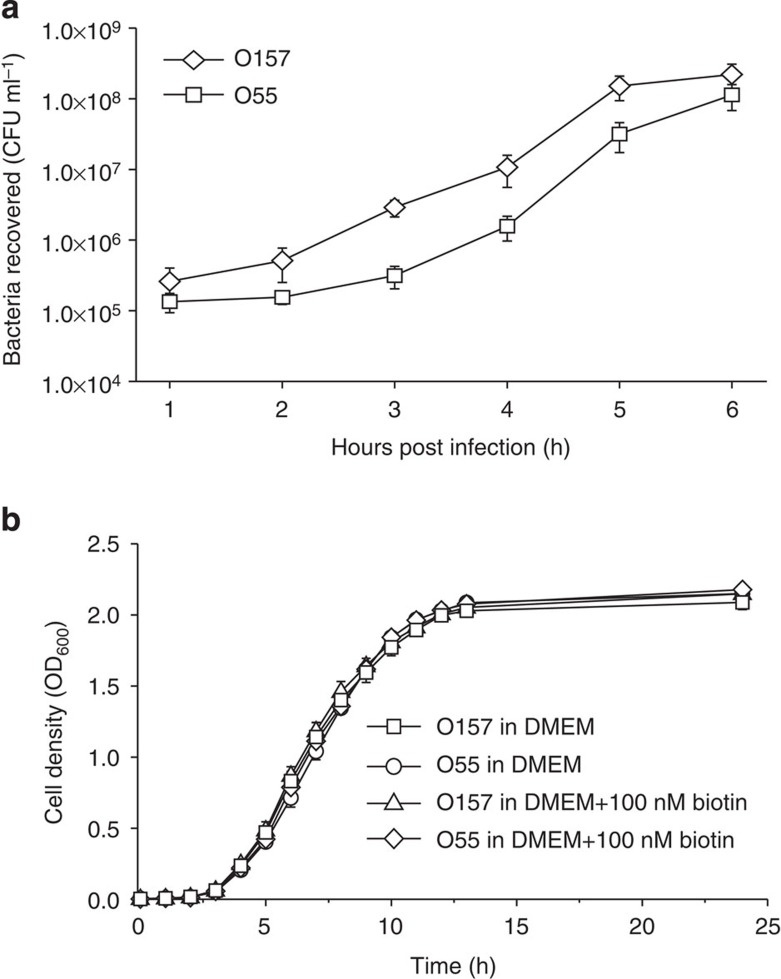
The adherence and growth of O157 and O55 *in vitro*. (**a**) The numbers of O157 and O55 cells recovered at 1–6 h post infection of HeLa epithelial cells. (**b**) The growth of O157 and O55 in DMEM supplemented with 0 or 100 nM biotin. Data are presented as means±s.d.; *n*=3.

HeLa infection experiments were carried out to compare the adherence abilities of O157 and O55 by incubating log-phase Dulbecco’s modified Eagle medium (DMEM)-grown bacterial cells with HeLa epithelial cells for 6 h. These assays were assessed at hourly intervals, and it was found that the number of HeLa-attached O157 cells was 1.92–9.20 times higher compared with O55, with the largest difference being observed at 3 h (Fig. 1a). The two strains exhibited similar growth rates in DMEM (Fig. 1b), and the total numbers of attached and non-attached cells were also similar between the two strains during the 6-h course of the infection (Supplementary Fig. 1), indicating that the increase in the number of O157 cells adhering to HeLa cells was not due to different growth rates. Because both strains resumed growth after 4 h of infection into HeLa cells, as indicated by the rapid increase in the total number of cells (Supplementary Fig. 1), a 3-h incubation period was selected as the optimal attachment time for transcriptome analysis and subsequent adherence assays.

### Transcriptional analysis of HeLa-attached O157 and O55

To investigate the molecular basis of the different adherence characteristics of O157 and O55, transcriptome analyses were carried out to reveal differences in global gene expression profiles between HeLa-attached O157 and O55 cells (DMEM-grown cells incubated with HeLa cells for 3 h) in comparison with free-living DMEM-grown cells. In total, 11,822,200–26,056,440 raw reads from different control and experimental groups were generated using Solexa/Illumina RNA-seq deep-sequencing analysis. After low-quality reads were removed, 10,599,805–23,525,548 reads from each library were considered for further analysis. There were 5,084,499 and 7,979,625 reads mapped to the host-cell genome in HeLa-attached O157 and HeLa-attached O55, respectively. The total number of reads mapped to the *E. coli* O157:H7 EDL933 genome and the *E. coli* O55:H7 CB9615 genome ranged from 1,347,940 to 20,979,282, and the mapping success rate was between 13.70% and 94.34% (Supplementary Table 1). A total of 5,397 genes were detected in O157 and 5,121 in O55, of which 4,194 were conserved in both strains. The differentially expressed genes in HeLa-attached O157 and O55 compared with DMEM-grown bacteria are shown in Supplementary Fig. 2. The changes in the expression of the conserved genes were also compared. The expression levels of 1,069 genes were either upregulated (517) or downregulated (552) in both HeLa-attached O157 and O55 (Supplementary Data 1). Of these genes, many virulence genes related to the initial stage of infection that are shared between the two strains, including LEE genes and other adhesin genes, showed similarly altered expression (Supplementary Data 2), which is expected, as both EHEC and EPEC utilize the same infection mechanism. Notably, the expression of LEE genes in both strains was sharply decreased (Supplementary Data 2). Verification through qRT–PCR revealed that the expression of LEE genes in both O157 and O55 was significantly increased in the first 2 h of infection, whereas it was almost completely inhibited from 3 h onwards (Supplementary Fig. 3a,b). This result is in agreement with previous findings showing that LEE genes are induced in the early phase of infection to promote adherence, then inhibited in the late phase after colonization is established as an energy-saving strategy^27^,^28^.

In contrast, the expression of 1,243 conserved genes was either altered in only one strain or showed opposite patterns in the two strains (Supplementary Data 1); these genes might be related to the different virulence features of the strains. Of particular interest, the transcription levels of all essential genes involved in biotin synthesis (*bioBFCD* operon and *bioA*) were significantly upregulated (5.70–33.43-fold) in HeLa-attached O157 but remained unchanged in HeLa-attached O55. This result was confirmed via qRT–PCR (Table 1). However, the transcription levels of *accA*, *accB*, *accC* and *accD,* encoding the biotin-dependent carboxylase (the only enzyme that requires biotin for its function in *E. coli*), were unchanged in both O157 and O55 (Table 1). Therefore, the upregulation of biotin synthesis in HeLa-associated O157 is unlikely to be related to the general metabolism of the bacteria, but rather, to a regulatory function related to adherence (see below).

### Biotin inhibits O157 adherence by controlling LEE genes

**Figure 2:**
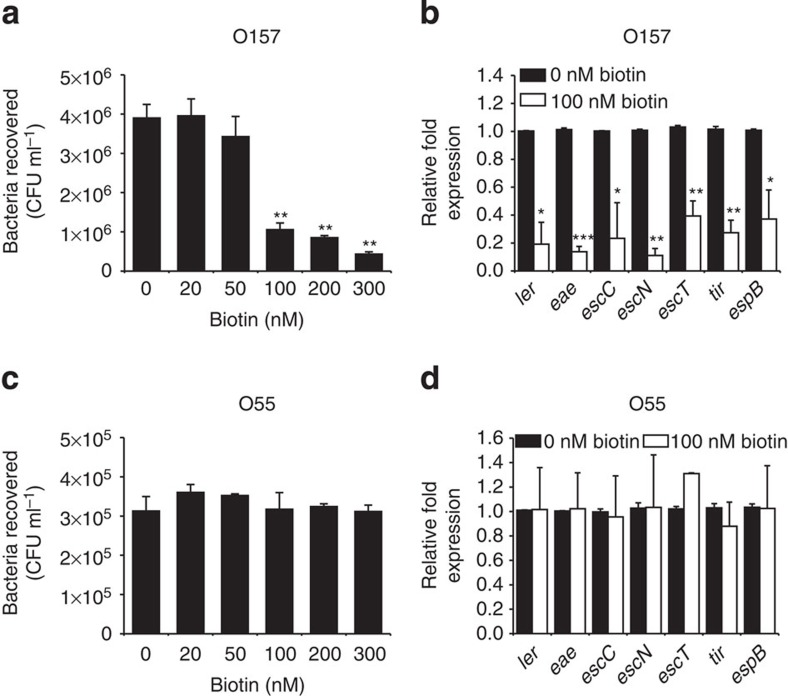
The effect of biotin on the bacterial adherence capacity and LEE gene expression. (**a**) Adhesion of O157 to HeLa cells in DMEM supplemented with different concentrations of biotin. (**b**) qRT–PCR was performed to measure the expression of LEE genes in O157 grown in DMEM supplemented with 0 or 100 nM biotin. (**c**) Adhesion of O55 to HeLa cells in DMEM supplemented with different concentrations of biotin. (**d**) qRT–PCR was used to measure the expression of LEE genes in O55 grown in DMEM supplemented with 0 or 100 nM biotin. Data are presented as means±s.d.; *n*=3. *P≤0.05; **P≤0.01; ***P≤0.001. All *P* values were calculated using Student’s *t*-test.

To further investigate whether biotin regulates O157 adherence, HeLa infection experiments were carried out in DMEM (biotin-free) supplemented with different concentrations of biotin. It was found that O157 adherence to HeLa cells was not affected by the presence of low levels of biotin (less than 50 nM) but decreased in the presence of high levels of biotin (above 100 nM; Fig. 2a). The same result was also obtained when Caco-2 intestinal epithelial cells were used instead of HeLa cells (Supplementary Fig. 4). The growth of O157 and O55 in DMEM was not affected by the presence of biotin (100 nM; Fig. 1b), indicating that the negative effect of biotin on O157 adherence was not due to different growth rates. The expression of representative LEE genes, including *ler* (the master regulator of LEE genes), *escT* (LEE1), *escC* (LEE2), *escN* (LEE3), *eae* (intimin, LEE5), *tir* (intimin receptor, LEE 5) and *espB* (LEE 4), was significantly reduced in the presence of high levels of biotin (100 nM) in O157, in comparison with the expression levels observed at 0 nM biotin (Fig. 2b). This result indicates that the negative effect of biotin on O157 adherence was due to the negative regulation of LEE genes by biotin. In contrast, the ability of O55 to adhere to HeLa or Caco-2 cells and express LEE genes were unaffected by the presence of biotin (Fig. 2c,d and Supplementary Fig. 4).

### BirA mediates biotin-induced O157 LEE gene repression

**Figure 3:**
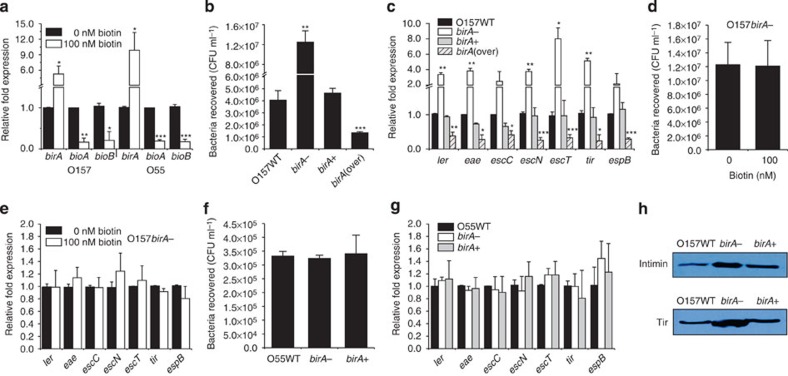
The effect of *birA* on the bacterial adherence capacity and the expression of biotin synthetic genes and LEE genes. (**a**) qRT–PCR quantification of changes in the expression of *birA*, *bioA* and *bioB* in O157 and O55 grown in DMEM supplemented with 0 or 100 nM biotin. (**b**) Adhesion of O157 WT, *birA*-, *birA*+ and *birA*(over) to HeLa cells. (**c**) qRT–PCR quantification of changes in the expression of LEE genes in O157 WT, *birA*-, *birA*+ and *birA*(over). (**d**) Adhesion of the O157 *birA*- strain to HeLa cells in DMEM supplemented with 0 or 100 nM biotin. (**e**) qRT–PCR quantification of the change in LEE gene expression in O157 *birA*- grown in DMEM supplemented with 0 or 100 nM biotin. (**f**) Adhesion of O55 WT, *birA*- and *birA*+ to HeLa cells. (**g**) qRT–PCR was performed to measure the expression of LEE genes in O55 WT, *birA*- and *birA*+. (**h**) Immunoblot analysis of intimin and its receptor, Tir, in O157 WT, *birA*- and *birA*+. Full blots are shown in Supplementary Fig. 9. *birA*-, *birA* mutant; *birA*+, *birA*-complemented strain; *birA*(over), *birA*-overexpression strain; WT, wild-type strain. Data are presented as means±s.d.; *n*=3. *P≤0.05; **P≤0.01; ***P≤0.001. All *P* values were calculated using Student’s *t*-test.

In *E. coli*, BirA is able to sense a high biotin status and repress the expression of biotin synthesis genes^20^. This ability was confirmed for both O157 and O55 by showing that the expression of *birA* increased, whereas the expression of *bioA* and *bioB* decreased in the presence of 100 nM biotin (Fig. 3a). Whether BirA is also involved in the biotin-induced repression of LEE genes in O157 was subsequently examined. Inactivation of *birA* in O157 resulted in an enhanced adherence capacity (Fig. 3b) and increased expression of LEE genes (Fig. 3c), and neither feature was affected by the presence of high levels of biotin (Fig. 3d,e). These changes could be restored to wild-type levels when a low-copy plasmid carrying the *birA* gene was introduced into the O157-Δ*birA* mutant strain (Fig. 3b,c), and overexpressing *birA* in O157 resulted in decreased bacterial adherence capacity (Fig. 3b) and LEE gene transcript levels (Fig. 3c). In contrast, inactivation of *birA* in O55 had no effect on bacterial adherence capacity, LEE gene transcription (Fig. 3f,g). Western blotting also revealed that the protein levels of intimin (encoded by *eae*) and its receptor, Tir, were significantly increased in the O157-Δ*birA* mutant strain compared with the O157 wild-type (WT) and complemented strains, whereas they remained unchanged in the O55-Δ*birA* mutant (Fig. 3h). These results confirmed that BirA acts as a negative LEE regulator in O157, but not in O55, and showed that the negative effect of biotin on O157 LEE genes is mediated by BirA.

The O157 genome was screened via ChIP-Seq analysis to reveal possible BirA-binding sites for the repression of LEE genes. A total of 6,591,200 and 6,011,222 raw reads were generated from the ChIP samples and mock ChIP samples. After low-quality reads were removed, the remaining 5,667,307 and 5,287,678 treated reads were mapped to the *E. coli* O157:H7 EDL933 genome. The number of uniquely mapped reads with no more than two mismatches from each library ranged from 4,669,886 to 5,091,616, yielding between 72.52 × and 79.22 × coverage of the reference genomes (Supplementary Table 2). Because of the depth of coverage, we used a low false discovery rate cutoff of 0.001% and a threefold enrichment to call BirA ChIP peaks. In addition to the known *bioO* site, 55 potential BirA-binding sites were identified, and 8 representatives were confirmed through ChIP-qPCR analysis (Supplementary Data 3). The presence of a large number of binding sites indicates that BirA plays multiple regulatory roles. However, no BirA-binding sites were found within the LEE region, indicating indirect regulation of LEE genes by BirA.

### Fur mediates the regulation of O157 LEE genes by BirA

**Figure 4:**
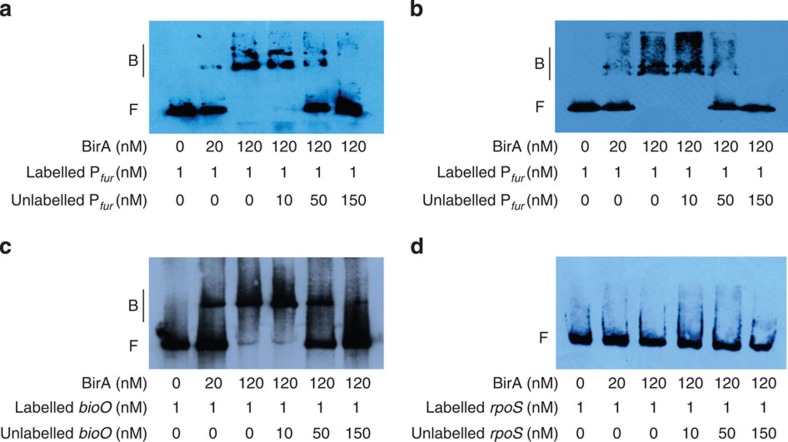
EMSAs and competition assays reveal the specific binding of BirA to the promoter region of *fur in vitro* for both O157 and O55. (**a**) O157, (**b**) O55, (**c**) positive control (*bioO*), (**d**) negative control (*rpoS*). The positions of the bound (denoted with ‘B’) and free (denoted with ‘F’) probe are shown on the left, and the concentrations of the probe and purified BirA are indicated at the bottom of each lane. Full blots are shown in Supplementary Fig. 10.

**Figure 5:**
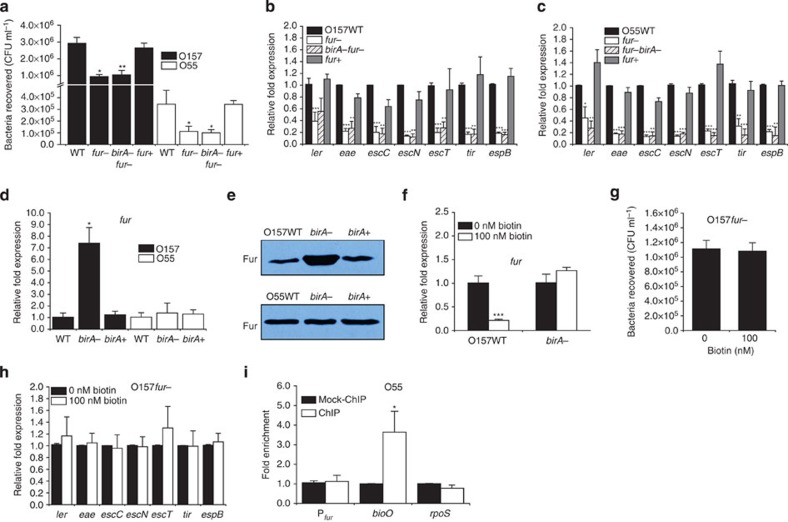
The regulation of adherence and LEE genes by BirA is mediated by *fur* in O157. (**a**) Adhesion of O157 and O55 WT, *fur*-, *fur*-*birA*- and *fur*+ to HeLa cells. (**b**) LEE gene transcript levels in O157 WT, *fur*-, *fur*-*birA*- and *fur*+. (**c**) LEE gene transcript levels in O55 WT, *fur*-, *fur*-*birA*- and *fur*+. (**d**) qRT–PCR quantification of changes in the expression of *fur* in O157 and O55 WT, *birA*- and *birA*+. (**e**) Immunoblot analysis of Fur in O157 and O55 WT, *birA*- and *birA*+. Full blot is shown in Supplementary Fig. 9. (**f**) qRT–PCR quantification of the change in expression of *fur* in O157 WT and *birA*- grown in DMEM supplemented with 0 or 100 nM biotin. (**g**) Adhesion of the O157 *fur*- to HeLa cells in DMEM supplemented with 0 or 100 nM biotin. (**h**) qRT–PCR quantification of the change in expression of LEE genes in the O157 *fur*- grown in DMEM supplemented with 0 or 100 nM biotin. (**i**) Fold enrichment of the *fur* promoter in O55 ChIP samples, as measured via ChIP-qPCR. *bioO* and *rpoS* are positive and negative controls, respectively. *birA*-, *birA* mutant; *birA*+, *birA*-complemented strain; *fur*-, *fur* mutant; *fur*-*birA*-, *fur birA* double mutant; *fur*+, *fur*-complemented strain; WT, wild-type strain. Data are presented as means±s.d.; *n*=3. *P≤0.05; **P≤0.01; ***P≤0.001. All *P* values were calculated using Student’s *t*-test.

Among the novel BirA-binding sites confirmed via ChIP-qPCR, one site was within the promoter region of *fur* (site 10 in Supplementary Data 3), which is a well-known global regulator^29^. The EMSAs and competition assays with O157 revealed that BirA binds specifically to the *fur* promoter and *bioO* (positive control), but not to *rpoS* (negative control) (Fig. 4a,c,d). The specific binding of BirA to the *fur* promoter was also observed in O55 (Fig. 4b). It was found that deletion of *fur* significantly reduced the ability of both O157 and O55 to adhere to HeLa cells and express LEE genes, and the ability to adhere could be restored to WT levels upon complementation with a functional copy of *fur* (Fig. 5a to c). These result indicated that Fur is a positive regulator of LEE genes in both strains. Although six putative Fur-binding sites (Supplementary Table 3) were identified within the O157 LEE region using bioinformatics, none were bound by Fur either *in vivo,* as indicated by ChIP-qPCR or *in vitro* in EMSAs; whereas Fur could bind to the *fepA* promoter (positive control) but not *rpoS* (negative control; Supplementary Table 3 and Supplementary Fig. 5), suggesting that the regulatory effect of Fur on LEE genes is indirect, which is also expected for O55 because the two strains share an identical LEE region. The lack of Fur binding to the promoter regions of other regulators involved in LEE regulation, including *hns*, *fis*, *ihfA*, *ihfB*, *grlAR*, *grvA*, *qseA*, *hha*, *gadE*, *hfq*, *etrA*, *eivF*, *rpoN* and *rpoS,* was also indicated through ChIP-qPCR (Supplementary Table 3). The regulator(s) of the indirect activation of LEE genes by Fur remains to be identified.

Additional *in vitro* experiments showed that both the transcription level and protein level of *fur* were significantly increased in the O157-Δ*birA* mutant, and both values could be restored to wild-type levels when a low-copy plasmid carrying the *birA* gene was introduced into the O157-Δ*birA* mutant (Fig. 5d,e). These results confirmed that BirA is a negative regulator of *fur*. Although the transcript level of *fur* in the O157 WT strain was significantly reduced by the presence of a high concentration of biotin (100 nM), the presence of biotin had no obvious effect on the expression of *fur* in the O157-Δ*birA* mutant (Fig. 5f). Neither the adherence capacity nor LEE gene expression of the O157-Δ*fur* mutant was affected by the presence of biotin (Fig. 5g,h), indicating that Fur is required in this biotin signalling virulence regulatory pathway. Furthermore, the observed adherence ability and the transcription levels of LEE genes were similar in the O157-Δ*fur* mutant and the O157-Δ*fur* Δ*birA* double mutant (Fig. 5a,b), indicating that the regulatory role of BirA on adherence and LEE gene expression in O157 is mediated by Fur.

Although the promoter region of *fur* in O55 is identical to that in O157, BirA is unable to bind to this region, as indicated through ChIP-qPCR analysis (Fig. 5i). The expression of *fur* at both the transcriptional and protein levels was also not affected by mutating *birA* in O55 (Fig. 5d,e and Supplementary Fig. 6), indicating that BirA shows no regulatory role in relation to *fur* in O55. It is possible that BirA access to the *fur*-binding site is prevented in O55 by the occupation of that region by other regulators.

### Negative effect of biotin on O157 adherence in mice

**Figure 6:**
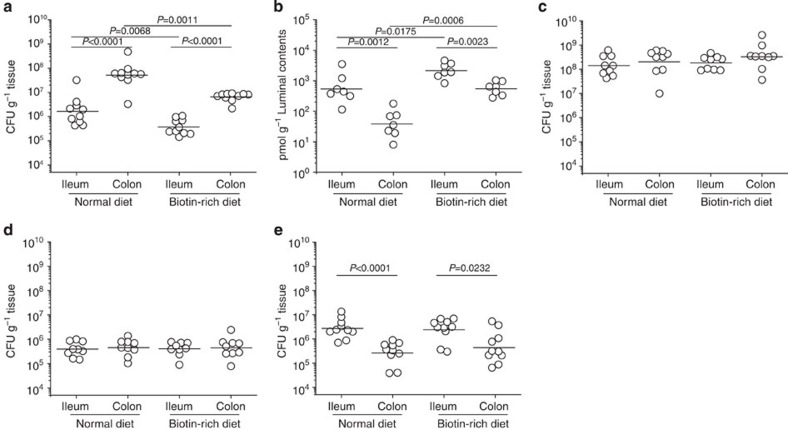
The effect of biotin on bacterial adherence capacity in the mouse intestinal tract. (**a**) Determination of the adherence capacity of O157 wild-type strain in the intestinal tract of mice maintained on a normal diet or a biotin-rich diet. (**b**) Quantification of biotin concentrations in the ileal contents and colonic contents obtained from mice fed a normal diet or biotin-rich diet. (**c**) Determination of the adherence capacity of the O157 *birA* mutant in the intestinal tract of mice maintained on either a normal diet or a biotin-rich diet. (**d**) Determination of the adherence capacity of the O157 *fur* mutant in the intestinal tract of mice maintained on either a normal diet or a biotin-rich diet. (**e**) Determination of the adherence capacity of O55 wild-type strain in the intestinal tract of mice maintained on a normal or biotin-rich diet. Each graph represents a typical experiment, with 7–10 mice in each group; the horizontal lines represent the geometric means. Statistical significance was assessed via the Mann–Whitney rank-sum test.

The effect of the biotin status of the host intestinal tract on O157 adherence was investigated using BALB/c mice maintained on a normal diet (standard mouse feed and sterilized water) or a biotin-rich diet (standard mouse feed and sterilized water containing 10 mM biotin) for 7 days. Under the normal diet, the number of bacteria that adhered to the colon (5.19 × 10^7^ colony-forming units (CFU) per gram (CFU g^−1^) tissue) was 31.5-fold higher than the number that adhered to the ileum (1.65 × 10^6^ CFU g^−1^ tissue; Fig. 6a), as expected. The biotin level in the ileal contents (538.6 pmol g^−1^ luminal content) was 14.3-fold higher than in the colonic contents (37.7 pmol g^−1^ luminal content; Fig. 6b). Under the biotin-rich diet, a similar difference in adherence was observed between the colon and ileum (Fig. 6a). However, the number of bacteria that adhered was 8.0 times (colon) and 4.4 times (ileum) lower than the number obtained from the same sites under the normal diet, whereas the biotin levels were increased 14.7 and 4.0 times in the respective luminal contents (Fig. 6a,b). Therefore, O157 adherence to the mouse intestinal tract was reduced by increased biotin levels in mice fed a high-biotin diet.

The molarity (nM) of biotin in mouse intestines was estimated based on the water content (ml) obtained by oven drying at 105 °C for 24 h, and the quantity of biotin (pmol) per gram of luminal content. The concentration of biotin was found to be approximately 450–700 nM in the ileal content (Supplementary Fig. 7); these concentrations are higher than the concentration (100 nM) determined by an *in vitro* experiment to induce repression of LEE genes. The biotin concentration was found to be 30–45 nM in the colonic content (Supplementary Fig. 7), which is less than the level for repression. Therefore, there was agreement between the *in vivo* and *in vitro* results for the levels of biotin that are required to induce repression.

The adherence of O157 to the mouse intestinal tract was enhanced by the deletion of *birA* and reduced by the deletion of *fur*, and the levels changed to a similar extent in mice fed either a normal diet or a biotin-rich diet (Fig. 6c,d), further confirming that the effect of biotin on O157 adherence is mediated by BirA and Fur. In contrast, the number of O55 that were recoverable from the mouse intestinal tract was much higher in the ileum (2.73 × 10^6^ CFU g^−1^ tissue) than in the colon (2.69 × 10^5^ CFU g^−1^ tissue) under the normal diet, and the feeding mice a biotin-rich diet had no obvious effect on O55 adherence (Fig. 6e).

### Negative effect of biotin on adherence is conserved in EHEC

**Figure 7:**
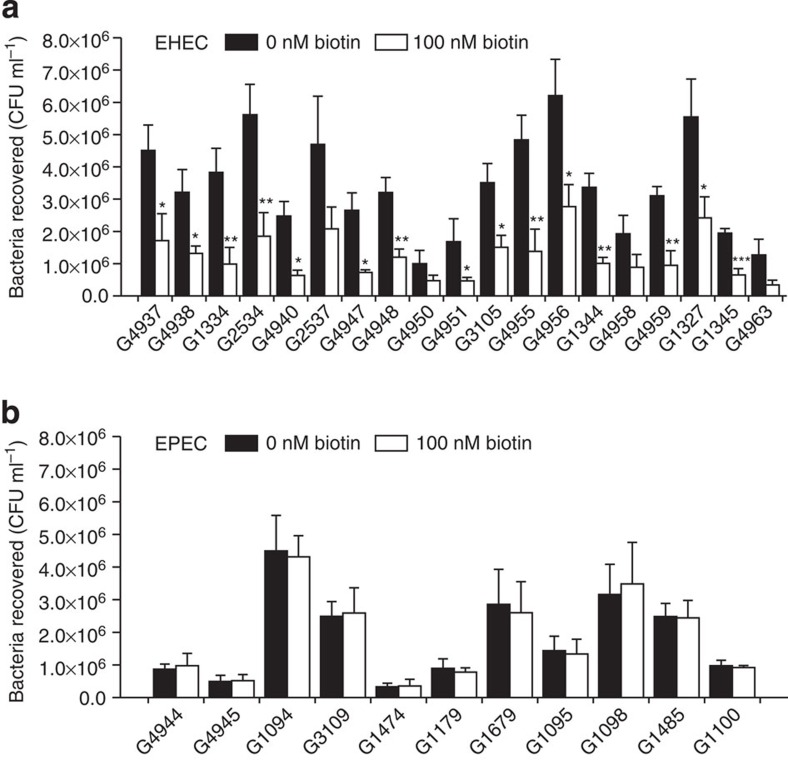
The effect of biotin on the adherence capacity of other EHEC and EPEC strains. (**a**) 19 EHEC strains, (**b**) 11 EPEC strains. Data are presented as means±s.d.; *n*=3. *P≤0.05; **P≤0.01; ***P≤0.001. All *P* values were calculated using Student’s *t*-test.

The presence of the biotin signalling regulatory pathway for the control of bacterial adherence was investigated using an additional 19 representative EHEC strains from nine serotypes and 11 EPEC strains from eight serotypes (Supplementary Table 4), isolated from different parts of the world, by carrying out HeLa infection experiments in the presence of 0 and 100 nM biotin. It was found that adherence to HeLa cells was significantly decreased in the presence of 100 nM biotin for all 19 EHEC strains (Fig. 7a). In contrast, biotin status had no obvious influence on the adherence abilities of the 11 EPEC strains (Fig. 7b).

## Discussion

The large intestine contains a high density of resident microflora, and pathogenic invaders must compete effectively for both limited nutrients and colonization sites^30^. To do so, EHEC has developed strategies for consuming mucus-derived carbohydrates more rapidly than the resident bacteria^31^ and for intimately adhering to host epithelial cells via the formation of A/E lesions^5^. The adoption of this BirA-mediated biotin signalling regulatory pathway to control the expression of LEE genes allows EHEC to recognize the large intestine for adhesion. Thus, it is an important pathway for the evolution of this pathogen as an invader of the large intestine. Invading the large intestine rather than the small intestine is apparently a better option for survival because the latter site is more hostile due to its lower pH, higher level of bile salts and more rapid movement of intestinal contents^32^.

**Figure 8:**
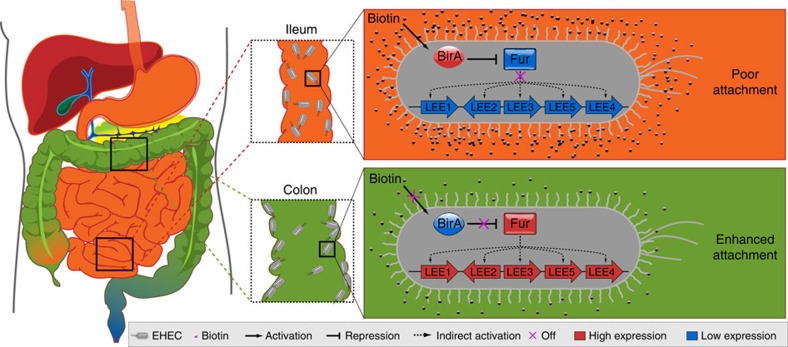
Model for the regulation of EHEC adherence by biotin in the human intestine. In the small intestine, the biotin level is high, and the expression of BirA is upregulated, repressing the expression of *fur*. The activation of LEE genes is inhibited, and adherence is therefore prevented. In the large intestine, the biotin level is low, and the expression of BirA is downregulated. The repression of *fur* is removed, allowing the activation of LEE genes by Fur to promote adherence.

A model for this regulatory pathway in EHEC O157 is proposed (Fig. 8). Briefly, when the bacterium enters the small intestine, where the biotin level is high, the expression of BirA is upregulated, repressing the expression of *fur*. Consequently, the activation of LEE genes by Fur is inhibited, and adherence is therefore prevented. This allows the bacteria to bypass the small intestine and enter the large intestine. Because the biotin level is low in the large intestine, the expression of BirA is downregulated, and the repression of *fur* is removed, allowing the activation of LEE genes by Fur to promote adherence. This pathway is incomplete in O55 due to the lack of a Fur response to BirA. Although biotin acts as a negative signalling molecule for EHEC adherence in the large intestine, the presence of other signals for EPEC adherence in the small intestine is predicted, which remain to be investigated. However, it is clear that EPEC and EHEC use different regulatory mechanisms to control the expression of LEE genes, leading to pathotype-specific colonization of the small and large intestines, respectively.

Current knowledge of the regulatory role of BirA is limited to the control of biotin synthesis associated with bacterial metabolism^20^,^22^. Here, we demonstrated, for the first time, that BirA is also involved in the regulation of a non-biotin-related process, specifically EHEC adherence, through modulating the expression of LEE genes. In both types of processes, BirA senses biotin levels to regulate the expression of the relevant genes. In contrast to the direct regulation of the *bio* operon by BirA, the regulation of LEE genes by BirA is indirect and is mediated by Fur. Fur has been shown to both directly repress and indirectly activate genes in response to iron and/or other environmental stimuli^29^. In EHEC, Fur is involved in the regulation of adhesin production, iron homeostasis, flagellum chemotaxis and acid and oxidative stresses^29^,^33^. Therefore, by interfering with Fur, BirA is able to affect other Fur-regulated virulence genes as well. More significantly, the regulatory role of BirA goes beyond its interaction with Fur, as we identified a large number of BirA-binding sites in the O157 genome. Collectively, these findings indicate that BirA is not only a specific regulator of biotin-related processes but also a global regulator that impacts many other functions.

Unfortunately, we were unable to elucidate the exact mechanism behind the regulatory role of Fur on O157 LEE gene expression and adherence. In addition to LEE genes, we also examined the possible regulatory role of Fur on other known adherence related genes reported in O157 and O55, including Efa1 adhesins (z4332), long polar fimbria (z4971, z5225), F9 fimbrial adhesin (z2200-z2203) and Paa adhesin (z2053), as well as *flhDC*, the Fur-regulated flagellar regulatory genes predicted *in silico*^34^, and two representative flagellum synthetic genes (*flgM* and *fliC*) by mutation and qRT–PCR analysis. However, none of these genes were found to be regulated by Fur (Supplementary Fig. 8). On the other hand, we also investigated known Fur-regulated LEE regulators, including the positive LEE regulators *rpoS*^35^ and *hns*^14^, as well as the negative regulators *fis*^36^ and *rpoN*^37^. However, none of these LEE regulators were found to be directly regulated by Fur as indicated by ChIP-qPCR analysis (Supplementary Table 3). We propose that the regulatory role of Fur on LEE genes could be indirectly mediated by those four regulators, although the presence of other Fur-regulated LEE regulators is also possible.

As expected, the expression of *fur* was induced at an early stage of HeLa infection and inhibited in the later stages, along with the expression of LEE genes, in the absence of external biotin (Supplementary Fig. 3c), indicating the presence of another *fur* regulator, most likely an activator. This activator must bind to the promoter region of *fur* to activate the gene. Some BirA can likely be sequestered from the *bioO* site to bind to the *fur* site when the bound activator is removed to inactivate *fur*, resulting in partial de-repression of the *bio* operon, explaining the increase in biotin synthesis observed in the late infection phase in O157. Therefore, BirA may also play a role in the repression of *fur* in the late infection stage of O157 in the absence of external biotin. The nature of the *fur* activator remains to be investigated. However, the identification of this novel regulatory pathway for LEE genes in O157 cannot explain the enhanced adherence of O157 to HeLa cells detected in the absence of external biotin, despite this observation being the initial driver of this study. More information is needed to clarify this point.

The weaker adherence to HeLa cells by DMEM-grown O55 compared with DMEM-grown O157 in the absence of biotin was apparently not related to biotin. Typical EPEC strains containing the EAF (EPEC adherence factor) plasmid usually have greater adherence abilities than atypical strains without the plasmid^38^. O55 is an atypical EPEC, which is likely to be one factor explaining its weaker adherence.

The number of sequencing reads mapped to the bacterial genome is lower in HeLa-attached O157 (4.92 M) and HeLa-attached O55 (1.35 M) compared with DMEM-grown samples (Supplementary Table 1); this difference could be a result of inefficient deletion of host-cell RNA from total RNA samples during library construction. However, the rates (%) of gene coverage by the sequencing reads were 87.92% in HeLa-attached O157 and 79.52% in HeLa-attached O55 (Supplementary Table 1). These values are within the range (70–95%) reported previously for bacterial transcriptome studies^39^,^40^,^41^. Although some of the differentially expressed genes might be missed and some values for the differences might be less accurate, the conclusions drawn in this study are not affected because all of the necessary genes involved in this regulatory pathway were further verified using qRT–PCR (Table 1 and Supplementary Fig. 3).

Both streptomycin mouse models and conventional mouse models have been widely used to investigate the adherence and pathogenesis of intestinal pathogens^42^. Colonization resistance is absent in streptomycin mice due to the lack of normal bowel flora, and this model is best suited to evaluating the relative colonization capacity of different strains via competitive infection studies (co-inoculated). In the present study, a conventional mouse model with an intact intestinal flora, which better reflects the typical gastrointestinal environment to which intestinal pathogens are exposed following ingestion, was used to examine the effect of biotin on the adherence capacity of EHEC and EPEC *in vivo*. Although this model require a high infection dose (10^9^ CFU), our results clearly indicated that biotin represses only the adherence of O157 in the mouse intestine and not that of O55.

Diarrhoeic diseases caused by EHEC are an important public health problem around the world. EHEC O157:H7 alone causes more than 73,000 illnesses, 2,100 hospitalizations and 60 deaths annually in the United States^43^. Several therapeutic strategies for diseases caused by EHEC have been developed, including the use of antibiotics and vaccination^44^,^45^. However, there is no established effective therapeutic strategy for treating EHEC infection and the use of antibiotics may be contraindicated, as bacterial lysis increases the release of Shiga toxin^43^. Our results revealed that high levels of biotin significantly reduced EHEC adherence in the mouse intestinal tract. Because biotin does not lyse cells and impose selection for resistance, it is attractive to propose the use of biotin as a potential antivirulence drug to prevent and control EHEC infection. For such applications, a slow-release form would ensure that the agent reaches the large intestine.

## Methods

### Bacterial strains, plasmids and growth conditions

The bacterial strains and plasmids used in this study are summarized in Supplementary Table 4. There are approximately 20 EHEC serotypes and 15 EPEC serotypes that are most commonly associated with human diseases^46^,^47^. In this study, we used 19 EHEC strains from nine serotypes and 11 EPEC strains from eight serotypes. The nine EHEC serotypes were O157:H7, O157:H-, O26:H11, O26:H-, O103:H2, O111:H8, O111:H-, O145:H28 and O70:H11, and the eight EPEC serotypes were O55:H7, O127:H-, O86:H34, O114:H2, O126:H-, O128:H2, O142:H6 and O145:H34. The strains were first verified through serotyping using polyclonal O-antigen and H-antigen antisera (Tianjin Biochip; # IM-EH004 and IM-EH001). A multiplex PCR assay was then performed to confirm the presence of intimin and Shiga toxin genes following the method of the STEC center ( http://www.shigatox.net/stec/cgi-bin/index). All of the EHEC strains used in this work contained the *eae* gene and at least one Shiga toxin gene, whereas all EPEC strains contained the *eae* gene but no Shiga toxin genes (see Supplementary Table 4 for details). Mutant strains were generated using the λ Red recombinase system^48^,^49^, and all strains were verified via PCR amplification and sequencing. To avoid lethality, we constructed the *birA* mutant in the presence of a plasmid-borne *birA* sequence from *Acinetobacter calcoaceticus,* following the method of Chakravartty and Cronan^50^. This heterologous BirA provides full biotin ligase function but has no regulatory function because it lacks the DNA-binding domain^51^. Complementary strains were constructed by cloning genes of interest into the pWSK129 or pTRC99A plasmid. The resulting constructs were then electroporated into the corresponding mutant strains. Strains for gene overexpression were constructed by cloning genes of interest into the pET28a expression vector; the resulting constructs were then electroporated into the *E. coli* BL21 strain. The primers used for the construction of the mutant strains and recombinant plasmids are described in Supplementary Table 5. All strains were maintained at −80 °C in Luria-Bertani (LB) broth with 20% glycerol and were grown overnight at 37 °C in LB broth when required. As necessary, antibiotics were added at the following final concentrations: ampicillin, 100 mg ml^−1^; chloramphenicol, 15 mg ml^−1^; and kanamycin, 50 mg ml^−1^.

### Sample preparation for RNA-Seq

HeLa cell infection was performed according to a previously described method with some modifications^52^,^53^. Briefly, HeLa cell cultures were grown at 37 °C under 5% CO_2_ until confluent. Three hours before infection, the HeLa cells were washed three times with pre-warmed phosphate-buffered saline (PBS), and the medium was replaced with fresh DMEM without antibiotics and fetal bovine serum. HeLa cell cultures were infected with exponential-phase O157 or O55 at a multiplicity of infection of 100:1 (refs 52, 54). After co-culturing with HeLa cells for 3 h, non-adherent bacteria were removed through extensive washing with pre-warmed PBS six times. The eukaryotic cells were lysed using lysis buffer (95% ethanol, 5% phenol and 0.1% SDS), and the adherent bacteria were collected as the experimental samples. As a control, logarithmic O157 or O55 was grown in fresh DMEM without HeLa cells for 3 h, and the DMEM-grown cultures were collected as control samples. These pelleted bacterial cells were used directly for RNA extraction. The assays were performed in three independent experiments.

### RNA preparation

Total RNA was isolated from samples using the TRIzol LS Reagent (Life; #10296-028) according to the manufacturer’s instructions. RNA samples were further purified using the RNeasy Mini Kit (Qiagen; #74104) and were treated with RNase-Free DNase I (Qiagen; #79254) to eliminate genomic DNA contamination. Host-cell RNA was depleted using the MICROB*Enrich* Kit (Ambion; #AM1901), and bacterial 23S and 16S rRNAs were subsequently depleted with the MICROB*Express* Bacterial mRNA Enrichment Kit (Ambion; #AM1905). RNA quality was determined using a Bioanalyzer (Agilent), and its quantity was assessed using a NanoDrop spectrophotometer (Thermo) after each manipulation step.

### RNA-Seq library construction and sequencing

The mRNA was fragmented using divalent cations under elevated temperatures. Random hexameric primers and the SuperScript Double-Stranded cDNA Synthesis Kit (Invitrogen; #11917-020) were used to synthesize double-stranded cDNA. The ends of these cDNA fragments were end repaired and A-tailed. The A-tailed fragments were then purified and ligated to Illumina adapters. These products were subsequently loaded into 2% low melting point agarose gels. The cDNA fragments between 200 and 250 bp in size were selected and purified using the Qiaquick Gel Extraction kit (Qiagen; #28704). After purification, a PCR assay using the obtained cDNA was performed, and the PCR products were purified using the Qiaquick PCR Purification Kit (Qiagen; #28104). Cluster formation, primer hybridization and single-end 36-cycle sequencing was performed according to the manufacturers’ recommended protocol. Three independent libraries were prepared for each of the RNA-Seq samples.

### Read mapping and statistics

The quality of all raw reads was assessed using the FastQC quality control tool with the default parameters, and the low-quality reads (Phred quality scores<20) were discarded. The treated reads from different libraries were then mapped to the *E. coli* O157:H7 EDL933 genome (NC_002655) and the *E. coli* O55:H7 CB9615 genome (NC_013941) using the Burrows-Wheeler Aligner with the following parameters: [-l 32] [-k 2] [-M 3] [-O 11] [-E 4]. The coverage at each gene was calculated, and the RPKM (reads per kilobase of coding sequence per million reads) method was used to quantify gene expression levels. The genes showing an RPKM≥10 were chosen for subsequent statistical analysis. In addition, genes that presented an RPMK<10 under one growth condition and an RPKM≥10 in the other condition were also accepted as candidates^55^. Differential gene expression was analyzed using the edgeR package^56^ with the default parameters. Genes exhibiting *P* values<0.05 and a threefold or greater difference in RPKM between two conditions were defined as differentially expressed.

### Chromatin immunoprecipitation

ChIP was performed as previously described^57^,^58^ with some modifications to the protocol. Inducible expression vectors carrying 3 × FLAG-tagged *birA* or *fur* were constructed and transformed into corresponding mutant strains. The bacterial cultures were grown to mid-logarithmic phase (OD_600_≈0.4) and were induced with 1 mM isopropyl-β-D-thiogalactoside for 30 min at 37 °C. To crosslink proteins to DNA, formaldehyde was added to a final concentration of 1%, and the samples were incubated at room temperature for 25 min. The cross-linking reaction was quenched by adding glycine to a final concentration of 0.5 M. The cross-linked cells were washed three times with ice-cold TBS and sonicated with 15 cycles of 30 s on/off at 95% power to generate DNA fragments of approximately 500 bp. Cell debris was removed, and the resulting supernatant was used as the cell extract for the immunoprecipitation of protein–DNA complexes using an anti-3 × FLAG antibody (Sigma; #F1804) and protein A magnetic beads (Invitrogen; #10002D) according to the manufacturer’s instructions. As a negative control, chromatin immunoprecipitation was performed on the other aliquot without the addition of antibodies. The RNA in the DNA sample was removed via incubation with RNaseA for 2 h at 37 °C, and proteins were then removed by incubation with proteinase K for 2 h at 55 °C. The DNA sample was subsequently purified with a PCR purification kit (Qiagen; #28104). All ChIP experiments were performed in three independent experiments.

### CHIP-Seq library construction and sequencing

ChIP DNA and mock ChIP DNA were end repaired, A-tailed and ligated to Illumina adapters. Size selection was performed to obtain a library with a median length of approximately 250 bp. After purification, a PCR assay was performed using Illumina genomic DNA primers 1.1 and 2.1, with 18 cycles of amplification. Cluster formation, primer hybridization, and single-end 36-cycle sequencing were performed according to the manufacturers’ recommended protocol. Three independent libraries were prepared for each of ChIP-Seq samples.

### Identification of protein-binding regions

The quality of the raw reads from the ChIP samples and mock ChIP samples was assessed using the FastQC quality control tool with the default parameters. The low-quality reads (Phred quality scores<20) were discarded. The treated reads were then aligned to the *E. coli* O157:H7 EDL933 reference genome allowing no more than two mismatches, using the Burrows-Wheeler Aligner with the following parameters: [-l 32] [-k 2] [-M 3] [-O 11] [-E 4]. The data file containing the genomic coordinates of the mapped reads was then transformed into BED file format. We applied a false discovery rate cutoff of 0.001 to identify BirA ChIP peaks using Site Identification from Short Sequence Reads^59^ with the following parameters: [-D 0.001] [-m 0.8] [-w 20] [-E 2]. Peaks identified by Site Identification from Short Sequence Reads with a relative enrichment of more than threefold were scored as potential BirA-binding sites.

### Bacterial adherence assays

Adherence assays were performed using a previously described method^60^. HeLa cells and Caco-2 cells were purchased from the Shanghai Institute of Biochemistry and Cell Biology of the Chinese Academy of Sciences (Shanghai, China). Before infection, HeLa cells were washed three times with pre-warmed PBS, and the medium was replaced with fresh DMEM without antibiotics and fetal bovine serum. Monolayers of cells were infected with an exponential-phase bacterial culture (10^8^ CFU per well), then incubated for 3 h at 37 °C in a 5% CO_2_ atmosphere. After incubation, the wells were washed six times with PBS to remove unattached bacteria. HeLa cells were then disrupted with 0.1% SDS at 37 °C for 5 min. Lysate dilutions were plated on LB agar, and the attachment efficiency was determined by counting the colony-forming units (CFU) per milliliter. For each assay, at least three independent biological replicates were performed.

### Quantitative RT–PCR

RNA samples were prepared using the TRIzol LS Reagent and were treated with RNase-Free DNase I to eliminate genomic DNA contamination. cDNA synthesis was conducted using the PrimeScript 1st Strand cDNA Synthesis Kit (Takara; #D6110A). qRT–PCR analysis was conducted using SYBR Green PCR master mix (Applied Biosystems; # 4367659) with the Applied Biosystems ABI 7300 sequence detection system. The 16S rRNA gene was used as a reference control to normalize differences in total RNA quantity among samples^61^. The relative difference in gene expression was calculated as the fold change using the formula 2^−ΔΔct^ (ref. 62). Expression change >2-fold and *P* values<0.05 were considered to be statistically significant. At least three biological replicates were performed for each qRT–PCR analysis.

### Western blot assays

Bacterial cultures were grown at 37 °C in DMEM to an OD_600_ of ≈1.0. The whole cells were harvested by centrifugation at 12,000*g* for 5 min, washed with PBS (pH 7.4), centrifuged, resuspended in ≈100 μl SDS–polyacrylamide gel electrophoresis solubilization buffer (normalized for OD_600_) and lysed at 100 °C for 10 min. Proteins were separated via 10% SDS–polyacrylamide gel electrophoresis and transferred to polyvinylidene difluoride membranes. The membranes were blocked with TBST (Tris-buffered saline with Tween 20) containing 5% non-fat milk for 1 h at room temperature. Incubations with the primary antibody anti-His (1:2,500 dilution, CWBIO; # CW0286) or anti-FLAG (1:2,500 dilution, Sigma; # F1804) and the secondary antibody goat anti-mouse-HRP (1:5,000 dilution, CWBIO; # CW0102) were then carried out for 1 h at room temperature. Blots were washed in TBST followed by detection with the ECL enhanced chemiluminescence reagent.

### Gel mobility shift assays

6 × His N-terminal-tagged BirA and 6 × His N-terminal-tagged Fur were purified using nickel columns (GE Healthcare; #17057501). BirA gel shift assays and competition assays were performed using the DIG Gel Shift Kit, 2nd Generation (Roche; #03353591910) according to the manufacturer’s instructions. For each assay, biotin and ATP were added to the binding buffer (1 mM Tris-HCl (pH 7.5); 0.1 mM EDTA; 0.2 mM dithiothreitol; 5 mM MgCl_2_; 10 mM KCl) at final concentrations of 20 μM and 1 mM, respectively^50^,^63^. Fur gel shift assays were performed using an EMSA Kit (Invitrogen; #E33075) according to the manufacturer’s instructions. For each assay, Mn^2+^ was added to the reaction buffer (offered by the kit) at a final concentration of 100 μM (refs 64, 65). Native polyacrylamide gels were used to separate free DNA probes and/or probe-protein complexes for each assay.

### Quantitative PCR

To measure the enrichment of potential Fur-binding targets or BirA-binding targets in the immunoprecipitated DNA samples, relative-abundance qPCR was performed with SYBR green mix. Relative target levels were calculated using the ΔΔCt method^62^. The results represent the average enrichment measured via qPCR in at least three biological replicate experiments.

### Mouse colonization experiments

All animal experiments were performed according to the standards set forth in the Guide for the Care and Use of Laboratory Animals^66^. The experimental protocols were approved by the Institutional Animal Care Committee at Nankai University. Six-week-old female BALB/c mice were maintained on either a normal diet (standard mice feed and sterilized water) or a biotin-rich diet (standard mice feed and sterilized water containing 10 mM biotin) provided *ad libitum* for 7 days. In each group, ten mice were orally infected by pipette feeding of 100 μl of PBS containing 10^9^ CFU of bacteria (O157WT, O157-Δ*birA*, O157-Δ*fur* and O55WT) Nal^R^ growing in logarithmic phase. The infected mice were anaesthetized in isoflurane and euthanized via cervical dislocation at 6 h after infection. The ileum and the colon were excised from the infected animals, and the luminal contents were then removed from each organ. Each portion of the intestine was washed vigorously with PBS three times and then weighed and homogenized in 0.5 ml of PBS. The homogenates were subsequently diluted, and the samples were plated on LB agar containing nalidixic acid (50 μg ml^−1^) to determine the number of CFU per gram of organ tissue.

### Quantification of biotin in mouse intestinal tracts

Six-week-old female BALB/c mice were fed a normal diet or biotin-rich diet *ad libitum* for 7 days. In each group, seven mice were euthanized, and the luminal contents were obtained. The luminal contents were homogenized in 1 ml of distilled water and centrifuged at 12,000 *g* at 4 °C for 10 min. The supernatant was diluted and used for biotin quantification. Biotin standards (0–1,000 pmol l^−1^) were freshly prepared in HEPES buffer (0.05 M, pH 7.0) from a stock solution of biotin (100 nM). Biotin quantification was performed via a streptavidin-binding assay, as described previously^67^. Biotin concentrations were interpolated on the standard curve and the results expressed as picomoles of biotin per gram luminal contents.

## Author contributions

L.W. and L.F. designed the research; B.Y. and F.W. performed the research; B.Y. and F.W. collected the data; L.W., L.F. and B.Y. analyzed the data; and L.W., L.F. and B.Y. wrote the paper.

## Additional information

**Accession codes:** All sequence data have been deposited in the NCBI SRA database under the accession codes SRR1619463, SRR1619464, SRR1619465 and SRR1641752 for the RNA-seq data and SRR1621223 and SRR1621224 for the ChIP-seq data.

**How to cite this article:** Yang, B. *et al.* Enterohemorrhagic *Escherichia coli* senses low biotin status in the large intestine for colonization and infection. *Nat. Commun.* 6:6592 doi: 10.1038/ncomms7592 (2015).

## Supplementary Material

Supplementary Information Supplementary Figures 1-10, Supplementary Tables 1-5 and Supplementary References

Supplementary Data 1 Genes with common and different changes in expression in O157 and O55, 3 h after incubation with HeLa cells

Supplementary Data 2 Up- or down-regulated LEE genes and adhesin genes in both HeLa-attached O157 and O55

Supplementary Data 3 Potential BirA-binding sites in the Escherichia coli O157:H7 EDL933 genome identified by ChIP-seq

## Figures and Tables

**Table 1:** Relative fold changes in the expression of biotin-related genes in O157 and O55 after 3 h of incubation with HeLa cells.

**Gene**	**Product/function**	**Fold change in O157**			**Fold change in O55**		
		Fold*	***P*** **value**	**Fold**†	Fold*	***P*** **value**	**Fold**†
*bioA*	Adenosylmethionine-8-amino-7-oxononanoate transaminase	11.30	1.95 × 10^−4^	10.97±1.05	1.26	0.99	1.23±0.42
*bioB*	Biotin synthase	13.24	4.92 × 10^−6^	3.43±0.29	−1.86	0.11	0.87±0.21
*bioC*	Biotin biosynthesis protein BioC	33.43	1.10 × 10^−6^	6.01±1.22	1.30	0.97	0.88±0.19
*bioD*	Dithiobiotin synthetase	5.70	3.89 × 10^−3^	5.63±2.24	ND	ND	1.26±0.37
*bioF*	8-Amino-7-oxononanoate synthase	9.30	3.88 × 10^−4^	4.28±0.65	ND	ND	1.07±0.69
*accA*	Acetyl-CoA carboxylase carboxyltransferase subunit alpha	1.34	0.78	1.47±0.43	2.12	0.39	1.20±0.32
*accB*	Acetyl-CoA carboxylase biotin carboxyl carrier protein subunit	−1.00	0.73	1.17±0.13	1.18	0.70	1.09±0.47
*accC*	Acetyl-CoA carboxylase biotin carboxylase subunit	1.30	0.83	1.06±0.28	1.16	0.69	0.91±0.26
*accD*	Acetyl-CoA carboxylase subunit beta	1.80	0.38	0.78±0.21	1.84	0.61	0.86±0.22

ND, not determined; -, downregulated.

*P* values were calculated using binomial test.

^*^Fold change measured via RNA-Seq.

^†^Fold change measured via qRT–PCR, the presented values are the mean±s.d. of three independent experiments.
