# Supplementary material for: Enterohemorrhagic Escherichia coli senses low biotin status in the large intestine for colonization and infection
Source: Nat Commun. 2015 Mar 20;6:6592. doi: 10.1038/ncomms7592 (PMC4382993; doi:10.1038/ncomms7592)
Supplement: Supplementary Information — Supplementary Figures 1-10, Supplementary Tables 1-5 and Supplementary References [file ncomms7592-s1.pdf]

Supplementary figures

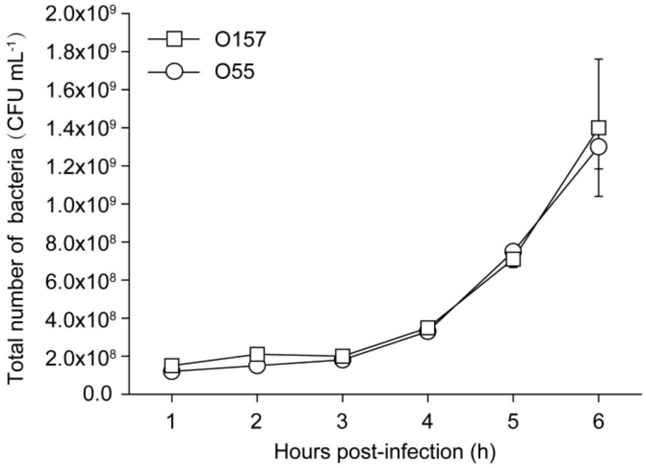

Supplementary Figure 1. The total numbers of attached and non-attached bacteria after 1 h to 6 h incubation of O157 or O55 with HeLa epithelial cells. Data are presented as means ± s.d.; n=3.

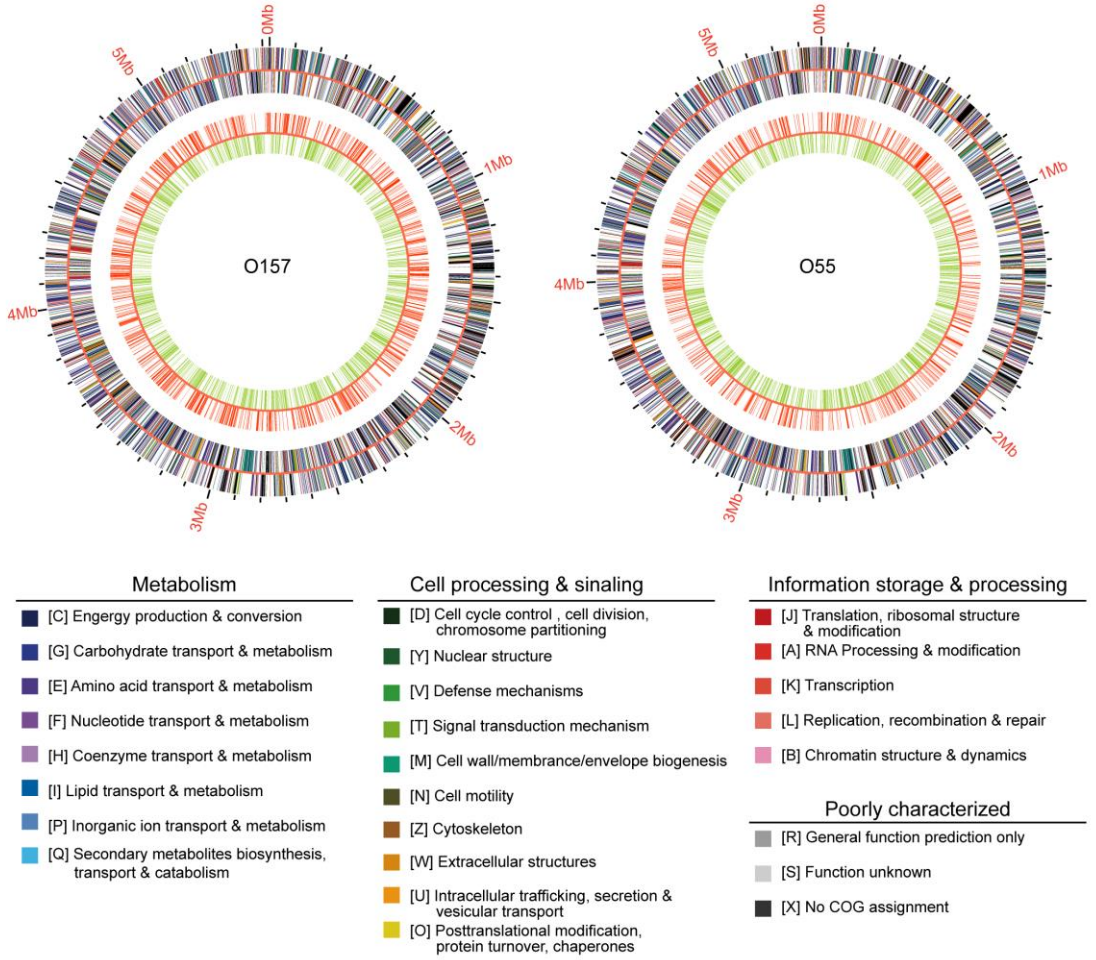

Supplementary Figure 2. Distribution of differentially expressed genes in the O157 and O55 genomes. From the outside in, circle 1 demonstrates the size in base pairs. Circles 2 and 3 indicate the CDSs in the clockwise and anti-clockwise directions, analyzed using the COG database (colors were assigned according the COG functional classes, see key). Circles 4 shows the position of the genes that are up-regulated 3 h after attachment of O157 or O55 to HeLa cells (colored orange). Circle 5 shows the position of genes that are down-regulated 3 h after attachment of O157 or O55 to HeLa cells (colored green).

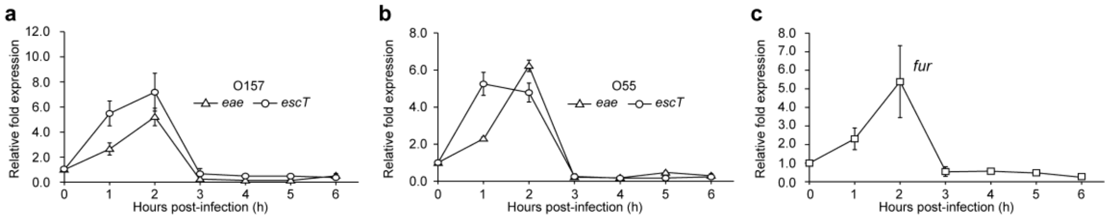

Supplementary Figure 3. (**a**) qRT-PCR quantification of the changes in expression of *eae* and *escT* in O157 incubated with HeLa cells from 1 to 6 h. (**b**) qRT-PCR quantification of the changes in the expression of *eae* and *escT* in O55 incubated with HeLa cells from 1 to 6 h. (**c**) qRT-PCR quantification of the changes in the expression of *fur* in O157 incubated with HeLa cells from 1 to 6 h. The expression of *eae*, *escT*, and *fur* in DMEM-grown O157 or O55 is represented as 1 (0 h), and all other values of expression from 1 h to 6 h are expressed relative to this value. Data are presented as means ± s.d.; n=3.

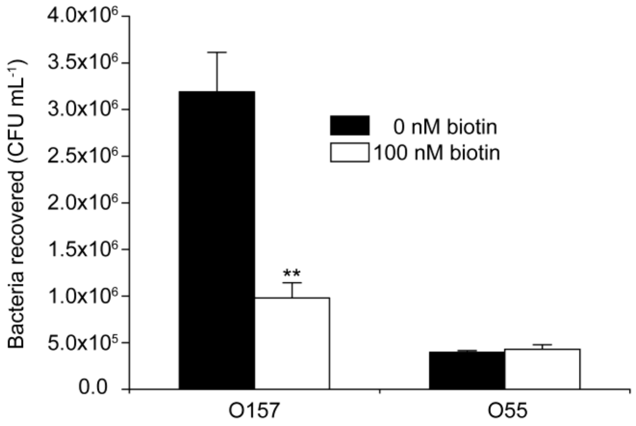

Supplementary Figure 4. Adherence of O157 and O55 to Caco-2 cells in the presence of 0 or 100 nM biotin. Caco-2 cells were grown to confluence in 6-well plates. Cell cultures were infected with exponential-phase O157 or O55 (10^8 bacteria/well) for 3 h at 37 °C in a 5% CO_2 atmosphere. The attachment efficiency was represented by the colony-forming units (CFU) per milliliter. Data are presented as means ± s.d.; n=3. ** P ≤ 0.01. All *P* values were calculated using Student's *t*-test.

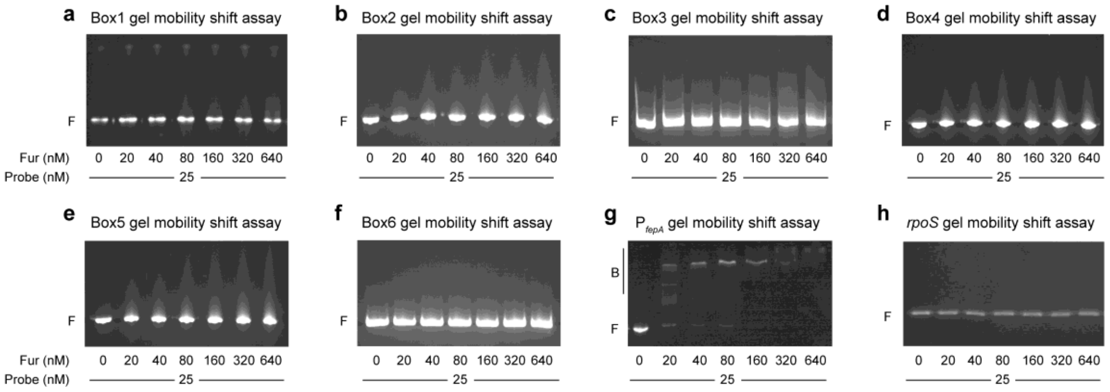

Supplementary Figure 5. The interaction of Fur with the predicted Fur boxes located within the LEE region. (**a-f**) Gel mobility shift analysis of Fur with the predicted Fur boxes (Box1 to Box6 in Supplementary Table 6) located within the O157 LEE region. (**g**) Gel mobility shift analysis of Fur with the *fepA* promoter region (positive control). The positions of the bound (denoted with “B”) and free (denoted with “F”) probe are shown on the left. (**h**) Gel mobility shift analysis of Fur with *rpoS* (negative control). For each assay, the concentrations of the probe and purified Fur are indicated at the bottom of each lane. Full gels are shown in Supplementary Figure 10.

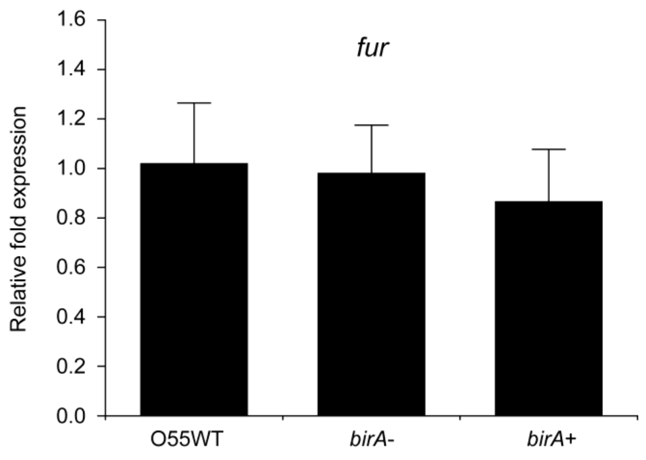

Supplementary Figure 6. Repetition of Figure 5d (O55). qRT-PCR quantification of the change in the expression of *fur* in the O55 wild-type strain and its *birA*-mutant and complemented strains. Data are presented as means ± s.d.; n=3.

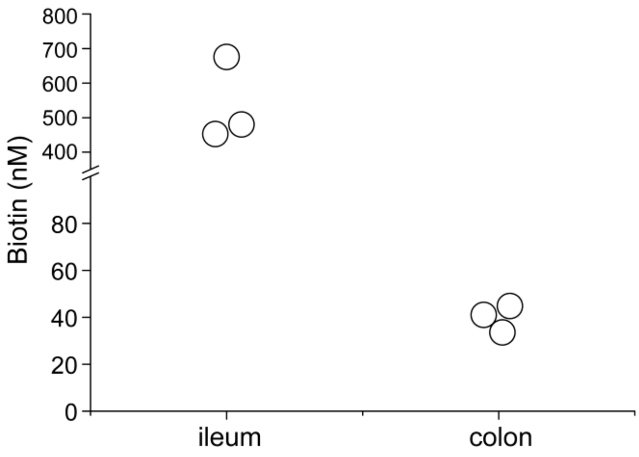

Supplementary Figure 7. Quantification of the molarity of biotin in the ileal content and colonic content obtained from mice fed a normal diet. The graph represents a typical experiment with 3 mice in each group.

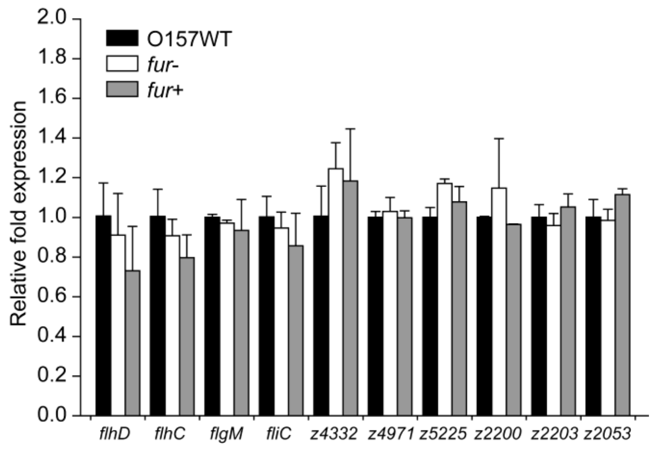

Supplementary Figure 8. qRT-PCR quantification of the changes in expression of *flhDC*, flagellum synthetic genes, and genes encoding initial adhesins in the O157 wild-type strain and its *fur*-mutant and complemented strains. Data are presented as means ± s.d.; n=3.

Fig. 3h

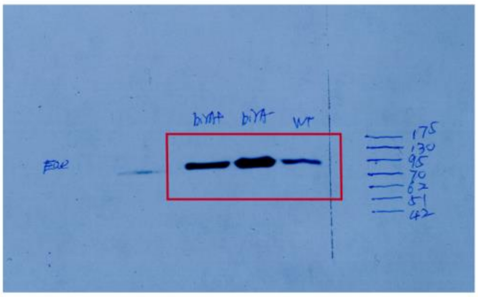

Fig. 3h

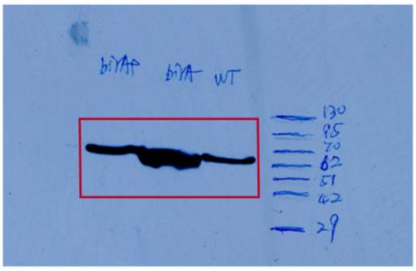

Fig. 5e

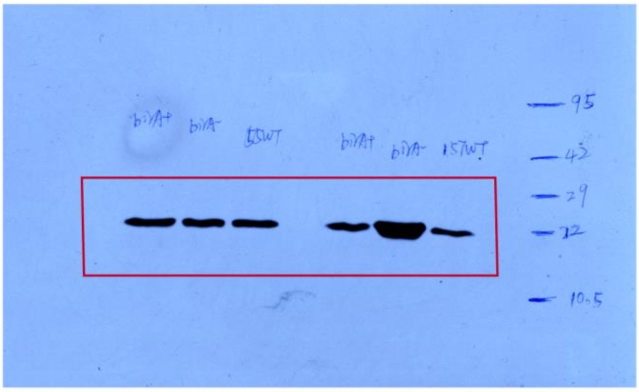

Supplementary Figure 9. Scans of the western blots. Boxes highlight lanes used in the figures.

Fig. 4a

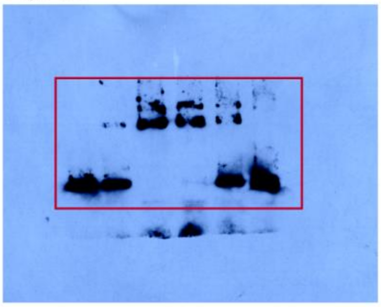

Fig. 4b

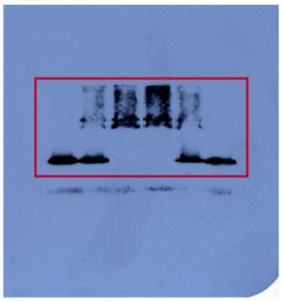

Fig. 4c

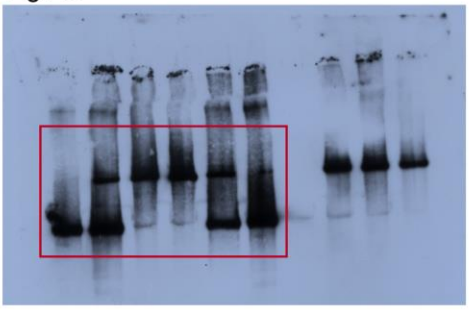

Fig. 4d

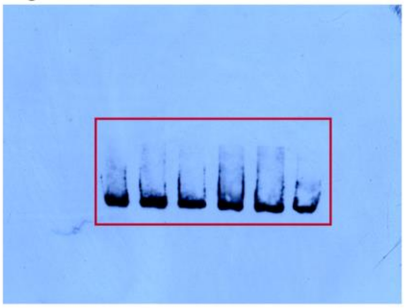

Supplementary Fig. 5a

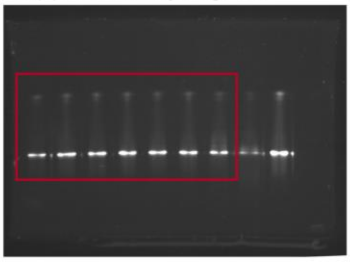

Supplementary Fig. 5b

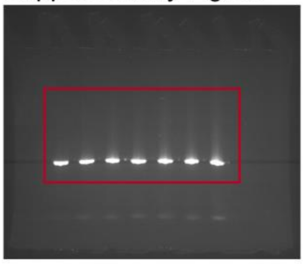

Supplementary Fig. 5c

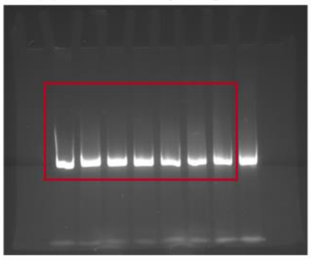

Supplementary Fig. 5d

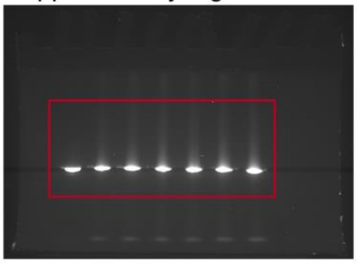

Supplementary Fig. 5e

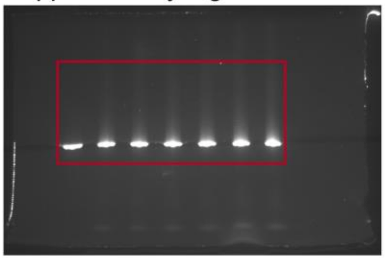

Supplementary Fig. 5f

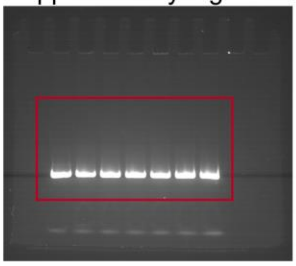

Supplementary Fig. 5g

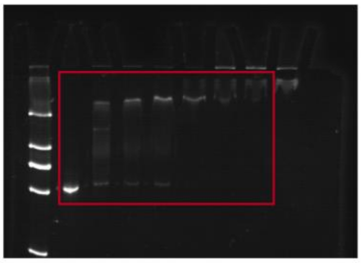

Supplementary Fig. 5h

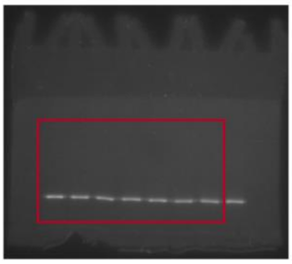

Supplementary Figure 10. Full blots and gels of the EMSAs. Boxes highlight lanes used in the figures.

Supplementary Tables

Supplementary Table 1. Statistics for the RNA-seq data sets

	DMEM-grown O157	HeLa-attached O157	DMEM-grown O55	HeLa-attached O55
Total number of reads	12,604,656	12,353,582	26,056,440	11,822,200
Average read length (bp)	74.20	78.34	74.56	76.13
Low-quality reads	1,186,530	1,126,642	2,530,852	1,222,395
Multi-mapped reads	336,973	773,344	136,363	68,528
Reads mapped to bacterial genome	9,916,754	4,917,044	20,979,282	1,347,940
Mapping success rate (%)	92.05	46.71	94.34	13.70
Reads mapped to host genome	/	5,084,499	/	7,979,625
Contamination rate with host RNA (%)	/	48.30	/	81.10
Gene coverage (%)	94.66	87.92	96.17	79.52

Supplementary Table 2. Statistics for the ChIP-seq data sets

	Mock-ChIP sample	BirA-ChIP sample
Total number of reads	6,011,222	6,591,200
Average read length (bp)	85.85	86.02
Low-quality reads	723,554	923,893
Multi-mapped reads	255,873	277,694
Reads mapped to bacterial genome	4,669,886	5,091,616
Mapping success rate	92.30%	93.27%
Coverage (fold)	72.52	79.22

Supplementary Table 3. The interaction of Fur with predicted Fur boxes located in the LEE region and the promoter regions of known LEE regulators in O157, as determined by ChIP-qPCR

Site_name	Putative Fur box	Start	End	Fold*	Reference
Box1†	AATAATAAATATGTTTAT	4662881	4662898	0.93 ± 0.10	This study
Box2	AATAAAAATAATTATATT	4670769	4670786	0.94 ± 0.41	This study
Box3	CATTATTATTTTCTATTAC	4673538	4673556	0.80 ± 0.19	This study
Box4	CATAAAGATAATAATCAT	4680198	4680215	0.75 ± 0.13	This study
Box5	TATTATAATTTTATTAT	4687072	4687088	0.94 ± 0.13	This study
Box6	CATATTCATAATAAATAAT	4689132	4689150	1.17 ± 0.18	This study
P_{fepA}	TATTATGATAACTATTTGCA	694097	694117	6.68 ± 1.63	[1]
rpoS	none			1.05 ± 0.17	This study
P_{hns}	none			0.96 ± 0.25	This study
P_{fis}	none			1.03 ± 0.19	This study
P_{ihfA}	none			0.84 ± 0.20	This study
P_{ihfB}	none			0.81 ± 0.18	This study
P_{grlR}	none			1.23 ± 0.22	This study
P_{grvA}	none			1.13 ± 0.42	This study
P_{qseA}	none			1.10 ± 0.57	This study
P_{hha}	none			1.20 ± 0.31	This study
P_{gadE}	none			0.70± 0.16	This study
P_{hfq}	none			0.71± 0.13	This study
P_{etrA}	none			0.63± 0.22	This study
P_{eivF}	none			0.79± 0.14	This study
P_{rpoS}	none			0.99± 0.23	This study
P_{rpoN}	none			0.86± 0.16	This study

[*], Fold enrichment measured by ChIP-qPCR, and values shown are the means ± SD of three independent experiments. [†], Using a bioinformatics method allowing no more than two mismatches compared with the standard Fur box (Fur binding sequences, N A T W A T N A T W A T N A T WA T; N= any nucleoside; W=A or T) [2], six putative Fur boxes (Box1 to Box6) were predicted within the O157 LEE pathogenicity island.

Supplementary Table 4. Strains and plasmids used in this study

**Strains**	**Genotype or description**	**Source or reference**
G2734	Wild-type EHEC O157:H7 EDL933	ATCC*
G2583	Wild-type EPEC O55:H7 CB9615	BfR†
H2164	*birA* deletion mutant in G2734 (pLW1629)	This work
H2257	*birA* deletion mutant in G2583 (pLW1629)	This work
H2165	*fur* deletion mutant in G2734	This work
H2258	*fur* deletion mutant in G2583	This work
H2259	*birA* and *fur* double deletion mutant in G2734	This work
H2404	*birA* and *fur* double deletion mutant in G2583	This work
H2400	H2164 containing pLW1693	This work
H2401	H2257 containing pLW1693	This work
H2205	H2164 containing pLW1630	This work
H2204	H2257 containing pLW1630	This work
H2402	H2165 containing pLW1694	This work
H2403	H2258 containing pLW1694	This work
H2212	H2165 containing pLW1633	This work
H2211	H2258 containing pLW1633	This work
H2206	DH5α containing pLW1631	This work
H2207	BL21 containing pLW1631	This work
H2213	DH5α containing pLW1634	This work
H2214	BL21 containing pLW1634	This work
H2261	H2164 containing pLW1658	This work
H2262	H2257 containing pLW1658	This work
H2264	H2165 containing pLW1659	This work
H2265	H2258 containing pLW1659	This work
H2405	G2734, intimin tagged with His tag	This work
H2406	H2164, intimin tagged with His tag	This work
H2407	H2400, intimin tagged with His tag	This work
H2408	G2734, Tir tagged with His tag	This work
H2409	H2164, Tir tagged with His tag	This work
H2410	H2400, Tir tagged with His tag	This work
H2411	G2734, Fur tagged with 3×FLAG tag	This work
H2412	H2164, Fur tagged with 3×FLAG tag	This work
H2413	H2400, Fur tagged with 3×FLAG tag	This work
H2414	G2583, Fur tagged with 3×FLAG tag	This work
H2415	H2257, Fur tagged with 3×FLAG tag	This work
H2416	H2401, Fur tagged with 3×FLAG tag	This work
G4937	EHEC O157:H7 (*eae*+, *stx*1+, *stx*2+)	[3]
G4938	EHEC O157:H7 (*eae*+, *stx*2+)	[4]
G1334	EHEC O157:H7 (*eae*+, *stx*1+, *stx*2+)	BfR

G2534	EHEC O157:H7 (*eae*+, *stx*1+, *stx*2+)	BfR
G4940	EHEC O157:H- (*eae*+, *stx*2+)	[5]
G2537	EHEC O157:H- (*eae*+, *stx*1+, *stx*2+)	BfR
G4947	EHEC O111:H8 (*eae*+, *stx*1+)	Lab collection
G4948	EHEC O111:H8 (*eae*+, *stx*1+, *stx*2+)	[6]
G4950	EHEC O111:H- (*eae*+, *stx*1+, *stx*2+)	Lab collection
G4951	EHEC O111:H- (*eae*+, *stx*1+, *stx*2+)	[7]
G3105	EHEC O111:H- (*eae*+, *stx*1+, *stx*2+)	Lab collection
G4955	EHEC O26:H11 (*eae*+, *stx*1+)	[4]
G4956	EHEC O26:H11 (*eae*+, *stx*1+)	[8]
G1344	EHEC O26:H11 (*eae*+, *stx*1+)	BfR
G4958	EHEC O26:H- (*eae*+, *stx*1+)	[9]
G4959	EHEC O26:H- (*eae*+, *stx*1+)	BfR
G1327	EHEC O103:H2 (*eae*+, *stx*1+)	BfR
G1345	EHEC O145:H28 (*eae*+, *stx*1+, *stx*2+)	BfR
G4963	EHEC O70:H11 (*eae*+, *stx*1+)	[10]
G4944	EPEC O55:H7 (*eae*+)	Aus‡
G4945	EPEC O55:H7 (*eae*+)	[11]
G1094	EPEC O127:H- (*eae*+)	Aus
G3109	EPEC O127:H- (*eae*+)	Lab collection
G1474	EPEC O86:H34 (*eae*+)	Aus
G1179	EPEC O114:H2 (*eae*+)	BfR
G1679	EPEC O126:H- (*eae*+)	Aus
G1095	EPEC O128:H2 (*eae*+)	Lab collection
G1098	EPEC O142:H6 (*eae*+)	Aus
G1485	EPEC O145:H34 (*eae*+)	BfR
G1100	EPEC O145:H34 (*eae*+)	Lab collection
**Plasmids**		
pKD3	Containing a chloramphenicol resistance cassette and the flipase recognition sites, CmR	[12]
pKD4	Containing a kanamycin resistance cassette and the flipase recognition sites, KmR	[12]
pKD46	Red recombination plasmid, ApR	[12]
pCP20	FLP expression plasmid, ApR, CmR	[12]
pET28a+	T7 expression vector, KmR	Novagen
pWSK29	Expression vector, ApR	[13]
pTRC99A	Expression vector, ApR	[14]
pACYC184	Expression vector, CmR, TcR	[15]
pLW1629	pACYC184 carrying *birA* from *Acinetobacter calcoaceticus* (AC), CmR	This work
pLW1693	pWSK29 carrying *birA*, ApR	This work
pLW1694	pWSK29 carrying *fur*, ApR	This work
pLW1630	pTRC99A carrying *birA*, ApR	This work

pLW1633	pTRC99A carrying *fur*, ApR	This work
pLW1631	pET28a carrying *birA*, KmR	This work
pLW1634	pET28a carrying *fur*, KmR	This work
pLW1658	pTRC99A carrying *birA*-3xFLAG, ApR	This work
pLW1659	pTRC99A carrying *fur*-3xFLAG, ApR	This work

[*], ATCC, American Type Culture Collection, Manassas, Virginia, USA. [†], BfR, Beutin Federal institute for Risk Assessment National Reference Laboratory for *Escherichia coli*, Berlin, Germany. [‡], Aus, School of Molecular and Microbial Biosciences, University of Sydney, Sydney, Australia.

Supplementary Table 5. Primers used in this study (5'-3')

Primers for gene mutation		
birA	F	GTGCATCAGGTGAAGTGAGCGCAGTGGAGACAATTTCGTGTAGGCTGGAGCTGCTTC
birA	R	CGCAAATAATTTGCAGGGGAGCTAATGCTCCCCTTTCATGGGAATTAGCCATGGTCC
fur	F	ATGAAGTGAACCGCTTAGTAACAGGACCAGATTCCGCGTGTAGGCTGGAGCTGCTTC
fur	R	GCCAACCCGCAGGTTGGCTTTTCTCGTTCAGACTGGCATGGGAATTAGCCATGGTCC
eae-his	F	TAATGTTAATACTCCAAATGTCTATGCGGTTTGTGTAGAACACCACCACCACCACCA CTAATAGGTGTAGGCTGGAGCTGCTTC
eae-his	R	ACTAAAACAATACATATTTTAGCCGGGGTGGTTATGGAAATGGGAATTAGCCATGGT CC
tir-his	F	TTCGAATAACCCACCAGCGCCGGGATCCCATCGTTTCGTCCACCACCACCACCACCA CTAATAGGTGTAGGCTGGAGCTGCTTC
tir-his	R	CCCCCTCCCTCCCTCTAAATAAAATGATTATGGATATATATGGGAATTAGCCATGGT CC
fur-3×flag	F	CGATTGCCGCGAAGATGAGCACGCGCACGAAGGCAAAGACTACAAAGACCATGACG GTG
fur-3×flag	R	GCCAACCCGCAGGTTGGCTTTTCTCGTTCAGACTGGCTTACGCCCCGCCCTGCCACTC A
Primers for gene cloning		
birA	F	CGGGATCCATGAAGGATAACACCGTGCC
birA	R	CCCAAGCTTTTCTTATTTTTCTGCACTACGCA
fur	F	CGGGATCCATGACTGATAACAATACCGCCCT
fur	R	CCCAAGCTTTTATTTGCCTTCGTGCGCGT
birA (AC)	F	CCCAAGCTTGCTAAATTACGAAAAACAGGC
birA (AC)	R	CGGGATCCGTGTTTCCGATATCAAGCCA
Primers for BirA-3×FLAG and Fur-3×FLAG (plasmid)		
birA	F	CATGCCATGGTCATGAAGGATAACACCGTGC
birA	R	CGGAATTCTTACTATTTATCGTCGTCATCTTTGTAGTCGATATCATGATCTTTATAATC ACCGTCATGGTCTTTGTAGTCTTTTTCTGCACTACGCAGGGATAT
fur	F	CATGCCATGGGCATGACTGATAACAATACCG
fur	R	CGGAATTCTTACTATTTATCGTCGTCATCTTTGTAGTCGATATCATGATCTTTATAATC ACCGTCATGGTCTTTGTAGTCTTTGCCTTCGTGCGCGTGCTCATCT
Primers for qRT-PCR		
rrsH	F	GAAAGCGTGGGGAGCAAAC
rrsH	R	ACATGCTCCACCGCTTGTG
ler	F	CAGGAAGCAAAGCGACTG
ler	R	ACCAGGTCTGCCCTTCTT
eae	F	GACGGTAGTTCACTGGACTTCTT
eae	R	TCGCCACCAATACCTAAACG
escC	F	GACCAAAATGTTGTCGTCCCA
escC	R	AGGTTACCGCTTCGCTCG
escN	F	AGGTTTTCTTGTTGCCTTTTGA

escN	R	TCTCCATTGGTCTGCCTATGC
escT	F	GCAATAGATGCGGCTGGAC
escT	R	TCGGCTTGTAATGGTAATATCTCG
tir	F	GATGTTCCTGGACTTCCTGTAA
tir	R	CAACAGAGGTCTCAACACCATT
espB	F	AAAACTCCTCGGCAAGATGG
espB	R	AATAATCCCGCCAACCAAAG
bioA	F	GGATGGTGAATGGGATGAGC
bioA	R	TCTCGTCGGCAATCAGCAA
bioB	F	GGAAGAATCCCCACGAACG
bioB	R	CCTAAACCCACAATGCCACC
bioC	F	AACATCGTATTCAGCCCATCAC
bioC	R	TCACGAACAATCACTCCCAAA
bioD	F	AACGGATACCGAAGTGGG
bioD	R	TGTTGTATCGCCTGTGCC
bioF	F	CGCCATCTTATCTACAGCACCA
bioF	R	GCAGCACCTCCAACAGACG
birA	F	ATGGCGGTAGTGTAACTGTGCT
birA	R	TCCAGACGCCAGAACATCG
accA	F	GCCATCCACAGCGTCCTT
accA	R	TTCTTTGGTTTCACGACCTTTT
accB	F	AAGAATCAGGCATCTCCGAACT
accB	R	CGTTGACTTTCTGACCCACTTC
accC	F	TTGCGTATTCTTCGTGCCTG
accC	R	AGCGTTCAACCTGCTCGG
accD	F	AAAAGCAACATTACTCCCACCC
accD	R	GGCTCAAGCTCGCTACCCA
fur	F	GTATCGTCACCCGCCACAA
fur	R	CGCAGTGACCGTAAAGATAGAGA
Z4332	F	GCATCAGAAGCCCGACTACG
Z4332	R	CATTTGCCAGACCAAGGAGC
Z4971	F	GAGATCGTGGATTCACCTTGC
Z4971	R	CGCCCGCTTCAGTGCTAA
Z5225	F	TACAGCCTTTAGTGGCGTCG
Z5225	R	TACCAGCGGCAGCATCG
Z2200	F	GCAGCCGAGGGTGATGAA
Z2200	R	GCCAGGCGAGAAGAACGA
Z2203	F	AGAGGCAGACGGCAGTATTCA
Z2203	R	GTCCAGTTTCCTTCCAGTCCAT
Z2053	F	TCTGCTCTGGCTATCATTCGG
Z2053	R	CAGGTCTGGTCAGGTCGTCAA
flhD	F	TAAATGAAGAAATGGCGACAACA
flhD	R	AGATCGTCAACGCGGGAAT

flhC	F	AGTGCCCGCAAGCAGAAG
flhC	R	TGGTGAGCGTGGGTAATAAAAT
fliC	F	TGCGAACGGTGTTGACGATG
fliC	R	GCCGCATCACCAGCAGAATA
flgM	F	GTATTGATCGCACTTCGCCTCT
flgM	R	ATATCACTGCTGCCGGGTTG
Primers for EMSAs		
P_{fur}	F	CTTTCTTTAGGGCGGTATTG
P_{fur}	R	TCAATGCCTGATGTGATGC
bioO	F	GCCCACCAGGACGACATA
bioO	R	GCTTTGCGGGCAGTATTT
Box1	F	GAAGAACTCGCACTCACA
Box1	R	AACAGAAGACTGGTTGCT
Box2	F	TAGTCGATAAACGGCTGAAC
Box2	R	TCCTGGTGTATAGCATGGC
Box3	F	GGATAATCAGTCCACCCAGG
Box3	R	TCCCGTATAGCGAAAGCC
Box4	F	CGATTTTGGAAACTACTGGG
Box4	R	GTTGGCGGCATCATTGT
Box5	F	TTGGCAGTAATGATAACAGAAA
Box5	R	GACATTAAACTTGATCCTGGC
Box6	F	GAGTCCATCATCAGGCACAT
Box6	R	TTTAAGGTGGTTGTTTGATGAA
P_{fepA}	F	TGTTTTATTCCTGCATTTTTG
P_{fepA}	R	TTCGTCATTCAGACGCTGC
rpoS	F	CTTCCAGTGTTGCCGCT
rpoS	R	CCCGTACTATTCGTTTGCC
Primers for ChIP-qPCR		
Site5	F	TGTATTGGCGTTGCTGTCG
Site5	R	GGCTTTAGTTCGCTGTGGGT
Site10	F	GCCACGCCTAAATGAAAACC
Site10	R	CGAAATTCTCAATGCCTGATGT
Site12	F	AAAATTAGCTCGCAACCTTCG
Site12	R	TGGGGCTTCTCCAAAACG
Site16	F	GGCAAGATCCAGCTCCGTC
Site16	R	CGTGCGTGTATTTGGTCAGGT
Site33	F	GGATGTCAGCCGCCGTAT
Site33	R	ATTACTTGCTTGCCAGACGC
Site39	F	TGTGGTCAGGGCTGGAAAA
Site39	R	GGTGAGGGAACCCAATACGT
Site40	F	TTTTCCCGCACCACGCTA
Site40	R	GCAGGCCATGCACCAGAG
Site44	F	GAAACTGACGCTGACCTACGC

Site44	R	GACGATAGTGCCTTCACCAAAC
Site45	F	ACGATGCCGAAATGCTGTC
Site45	R	CGCAAGGTACTCGCCATAAAA
rpoS	F	GTTATCGCAGGGAGCCACA
rpoS	R	TTTTACCACCAGACGCAAGTTA
Box1	F	CGAAGAACTCGCACTCACATTA
Box1	R	AACAGAAGACTGGTTGCTGGAG
Box2	F	CACGTAGTCGATAAACGGCTGA
Box2	R	TTAATTGGTTTTCTTTGGCTGTG
Box3	F	TAATCAGTCCACCCAGGTACGA
Box3	R	GGTGCCAAGAGTTATTCCCGTA
Box4	F	CACGATTTTGGAAACTACTGGG
Box4	R	TGTTGGCGGCATCATTGTA
Box5	F	GCTGTGAGCCAATGGTCATT
Box5	R	CATTAAACTTGATCCTGGCGAG
Box6	F	CGCAGTCGCTTTGCTTCC
Box6	R	GGTCCTTCCTGATAAGGTCGC
P_{hns}	F	AGCATTTCTTCCAGCGTTTCA
P_{hns}	R	GCGGCACAAAATAAAGAACAAT
P_{fepA}	F	CATCAACACGACGCATCCC
P_{fepA}	R	GACCTCAAACATTTCGTCATTCA
P_{fis}	F	TGGTATCTCCAGGAACACGCT
P_{fis}	R	TTAACCGAGTCACGCAGGG
P_{ihfA}	F	CATTTGGCGACGGTAGCG
P_{ihfA}	R	GTAAGGGTGTTGCGGAGGG
P_{ihfB}	F	GGTTTCGTCCTGTAATCAAGCA
P_{ihfB}	R	TGGGTGGCAAGTCTTTCTATCA
$P_{grlR/A}$	F	AAGCAATATCAAGAATAATGGAGACC
$P_{grlR/A}$	R	AAAACAATCGGAAGCATAACAGTAA
P_{grvA}	F	TTACTCAGAAAATAAAGGCAAGAACA
P_{grvA}	R	CCTATAACGGGGATACGCAGTT
P_{qseA}	F	GAGCAGGCGTTATTATTTTCAGC
P_{qseA}	R	GCGTTTTAGTCGTTCCATTATTCA
P_{hha}	F	CCTGACTTCCGCCATTTCTG
P_{hha}	R	TGAGCATATTGCGACCTTCG
P_{gadE}	F	TATGGAGTGCGTGATGGATAAAT
P_{gadE}	R	ACTGCCAAAAGCCCTGTAAAA
P_{hfq}	F	TACAGGTTGTTGGTGCTATCGC
P_{hfq}	R	GATTGCCCCTTAGCCATTCTC
P_{etrA}	F	CTATTCCCTCAACGTTAAGCAAAT
P_{etrA}	R	GTGAAGTAGACATCACTGGAAAAGAC
P_{eivF}	F	TTTCCCCGTAAGAACCACATTT
P_{eivF}	R	TGGACAGGATGACGTACTAGTGTAAG

P*rpoS*	F	CAGAAATGTTGGCAAGAATGGA
P*rpoS*	R	GGTATCGCCGCAGCAAGTAT
P*rpoN*	F	AAACGCATCATTGAGCACCTG
P*rpoN*	R	ACGTCGCAAACTTCTACCCTATC
Primers for EHEC/ EPEC identification		
mdh	F	AGGCGCTTGCACTACTGTTA
mdh	R	AGCGCGTTCTGTTCAAATG
eae	F	ATTATGGAACGGCAGAGGTTAAT
eae	R	ATCCCCATCGTCACCAGAGG
stx1A	F	CATTCGCTCTGCAATAGGTA
stx1A	R	AACTCGCGATGCATGATGA
stx2A	F	TATCTGGCGTTAATGGAGTT
stx2A	R	CCTGTCGCCAGTTATCTGAC

Supplementary References

1. de Lorenzo, V., Wee, S., Herrero, M. & Neilands, J. B. Operator sequences of the aerobactin operon of plasmid ColV-K30 binding the ferric uptake regulation (*fur*) repressor. *J Bacteriol*. **169,** 2624-2630 (1987).
2. Escolar, L., Perez-Martin, J. & de Lorenzo, V. Binding of the *fur* (ferric uptake regulator) repressor of *Escherichia coli* to arrays of the GATAAT sequence. *J Mol Biol*. **283,** 537-547 (1998).
3. Kurioka, T., Yunou, Y. & Kita, E. Enhancement of susceptibility to Shiga toxin-producing *Escherichia coli* O157:H7 by protein calorie malnutrition in mice. *Infect Immun*. **66,** 1726-1734 (1998).
4. Gansheroff, L. J., Wachtel, M. R. & O'Brien, A. D. Decreased adherence of enterohemorrhagic *Escherichia coli* to HEp-2 cells in the presence of antibodies that recognize the C-terminal region of intimin. *Infect Immun*. **67,** 6409-6417 (1999).
5. Bielaszewska, M., *et al.* Isolation and characterization of sorbitol-fermenting Shiga toxin (Verocytotoxin)-producing *Escherichia coli* O157:H- strains in the Czech Republic. *J Clin Microbiol*. **36,** 2135-2137 (1998).
6. Bopp, C. A., Greene, K. D., Downes, F. P., Sowers, E. G., Wells, J. G. & Wachsmuth, I. K. Unusual verotoxin-producing *Escherichia coli* associated with hemorrhagic colitis. *J Clin Microbiol*. **25,** 1486-1489 (1987).
7. Russmann, H., *et al.* Genotyping of Shiga-like toxin genes in non-O157 *Escherichia coli* strains associated with haemolytic uraemic syndrome. *J Med Microbiol*. **42,** 404-410 (1995).
8. Reid, S. D., Betting, D. J. & Whittam, T. S. Molecular detection and identification of intimin alleles in pathogenic *Escherichia coli* by multiplex PCR. *J Clin Microbiol*. **37,** 2719-2722 (1999).
9. Reid, S. D., Herbelin, C. J., Bumbaugh, A. C., Selander, R. K. & Whittam, T. S. Parallel evolution of virulence in pathogenic *Escherichia coli*. *Nature*. **406,** 64-67 (2000).
10. Toma, C., Higa, N., Iyoda, S., Rivas, M. & Iwanaga, M. The long polar fimbriae genes identified in Shiga toxin-producing *Escherichia coli* are present in other diarrheagenic *E. coli* and in the standard *E. coli* collection of reference (ECOR) strains. *Res Microbiol*. **157,** 153-161

(2006).

11. Escobar-Paramo, P., Clermont, O., Blanc-Potard, A. B., Bui, H., Le Bouguenec, C. & Denamur, E. A specific genetic background is required for acquisition and expression of virulence factors in *Escherichia coli*. *Mol Biol Evol*. **21,** 1085-1094 (2004).

12. Datsenko, K. A. & Wanner, B. L. One-step inactivation of chromosomal genes in *Escherichia coli* K-12 using PCR products. *Proc Natl Acad Sci U S A*. **97,** 6640-6645 (2000).

13. Wang, R. F. & Kushner, S. R. Construction of versatile low-copy-number vectors for cloning, sequencing and gene expression in *Escherichia coli*. *Gene*. **100,** 195-199 (1991).

14. Amann, E., Ochs, B. & Abel, K. J. Tightly regulated tac promoter vectors useful for the expression of unfused and fused proteins in *Escherichia coli*. *Gene*. **69,** 301-315 (1988).

15. Chang, A. C. & Cohen, S. N. Construction and characterization of amplifiable multicopy DNA cloning vehicles derived from the P15A cryptic miniplasmid. *J Bacteriol*. **134,** 1141-1156 (1978).
